# Genome-wide multimediator analyses using the generalized Berk–Jones statistics with the composite test

**DOI:** 10.1093/bioinformatics/btad544

**Published:** 2023-09-04

**Authors:** En-Yu Lai, Yen-Tsung Huang

**Affiliations:** Institute of Statistical Science, Academia Sinica, Nankang, Taipei 11529, Taiwan; Institute of Statistical Science, Academia Sinica, Nankang, Taipei 11529, Taiwan

## Abstract

**Motivation:**

Mediation analysis is performed to evaluate the effects of a hypothetical causal mechanism that marks the progression from an exposure, through mediators, to an outcome. In the age of high-throughput technologies, it has become routine to assess numerous potential mechanisms at the genome or proteome scales. Alongside this, the necessity to address issues related to multiple testing has also arisen. In a sparse scenario where only a few genes or proteins are causally involved, conventional methods for assessing mediation effects lose statistical power because the composite null distribution behind this experiment cannot be attained. The power loss hence decreases the true mechanisms identified after multiple testing corrections. To fairly delineate a uniform distribution under the composite null, Huang (Genome-wide analyses of sparse mediation effects under composite null hypotheses. *Ann Appl Stat* 2019a;13:60–84; AoAS) proposed the composite test to provide adjusted *P*-values for single-mediator analyses.

**Results:**

Our contribution is to extend the method to multimediator analyses, which are commonly encountered in genomic studies and also flexible to various biological interests. Using the generalized Berk–Jones statistics with the composite test, we proposed a multivariate approach that favors dense and diverse mediation effects, a decorrelation approach that favors sparse and consistent effects, and a hybrid approach that captures the edges of both approaches. Our analysis suite has been implemented as an R package MACtest. The utility is demonstrated by analyzing the lung adenocarcinoma datasets from The Cancer Genome Atlas and Clinical Proteomic Tumor Analysis Consortium. We further investigate the genes and networks whose expression may be regulated by smoking-induced epigenetic aberrations.

**Availability and implementation:**

An R package MACtest is available on https://github.com/roqe/MACtest.

## 1 Introduction

Investigating the possible consequences of an event is a matter of human instinct. Such investigations are conducted across various disciplines, such as to determine whether smoking changes gene expression profiles, a gene mutation leads to cancer, or air pollution influences lung function. Furthermore, merely knowing the cause and the corresponding effect is insufficient. To decipher the mechanisms that relate a cause to its consequences, we included intermediate variables in an analysis model, namely *mediators* (MacKinnon 2008), and conduct *mediation analyses* to quantify or evaluate the mediation effects. Mediation analysis is a statistical method that was first proposed by Baron and Kenny (1986) in the context of a single-mediator model. However, there usually exists a group of mediators involved in the mechanism that links exposure to an outcome; in the case of an aforementioned example, smoking may cause changes in epigenetic patterns, such as DNA methylation, and these modified methylation patterns cause corresponding changes in gene expression. The use of multiple mediators is thus an established methodology (Boca *et al.* 2014, VanderWeele and Vansteelandt 2014, Sampson *et al.* 2018), and some studies have further developed the methods for analyzing high-dimensional mediators (i.e. the number of potential mediators exceeds the sample size; Huang and Pan 2016, Huang 2019b, Zhou *et al.* 2020).

With the advent of high-throughput technologies, we begin to evaluate genes or proteins in genome-scale analyses instead of examining biomolecules one by one. Testing thousands of proteins or genes at once becomes a regular task for genome-wide studies; hence the need to accommodate multiple testing problems also emerges (Rice *et al.* 2008). One popular correction approach is to control the false discovery rate (FDR) of the genome-wide *P*-values (Benjamini and Hochberg 1995), but estimating FDR appropriately relies on the *P*-values being uniformly distributed under the null hypothesis. When we consider hypothesis testing for mediation effects, conventional methods tend to be conservative because of the composite null behind mediation. The power loss is especially conspicuous when only a few genes or proteins are causally involved, and it decreases the likelihood of identifying true mechanisms after FDR adjustment. Studies have compared the type I error rate and statistical power of single-mediator models (MacKinnon *et al.* 2002), developed efficient procedures for testing marginal mediation effects (Zhao *et al.* 2014), and constructed a series of single-mediator models to enable the assessment of element-wise and marginal mediation effects in a multiple-mediator model (Huang and Pan 2016). However, the discussion of testing mediation effects in genome-scale analyses (i.e. with the consideration of the following multiple testing correction) is very limited.

The following sections are organized as below, from Sections 2.1–2.3 we first introduce the composite test for single-mediator analyses proposed by Huang (2019a); the test is able to delineate uniformly distributed *P*-values under the composite null in a genome-wide analysis. Then, from Sections 2.4–2.4.2 we present a novel composite test for multimediator analyses; we use generalized Berk–Jones (BJ) statistics for testing the effects associated with multiple mediators, and we propose two frameworks (i.e. the decorrelation approach and the multivariate approach) for testing the mediation effect with the composite test. Section 3.1 describes numerous simulations to demonstrate that the composite test outperforms the other two tests when only a few mechanisms are causally involved. Finally, Section 3.2 details the application of our methods to The Cancer Genome Atlas Lung Adenocarcinoma (TCGA-LUAD) and the Clinical Proteomic Tumor Analysis Consortium Lung Adenocarcinoma (CPTAC-LUAD) datasets to demonstrate the utility of our testing procedures.

## 2 Materials and methods

### 2.1 Single-mediator model

Here, we introduce the notation of single-mediator models in genome-scale analyses. Let *S* be the exposure, *M* be the mediator, *Y* be the outcome, and **X** be the covariates of an individual. Consider a *genome-wide experiment* E that contains *g* possible mechanisms to be verified, denoted as $E=\{G^{i}|i\in 1,\ldots ,g\}$. For i∈{1,…,g}, the *i*-th *causal graph* (i.e. the graphical presentation of the *i*-th possible mechanism) is defined as a directed graph $G^{i}=\{V^{i},A^{i}\}$, where $V^{i}=\{S,M^{i},Y^{i}\}$ denotes the set of vertices, $A^{i}=\{a(S,M^{i}),a(M^{i},Y^{i}),a(S,Y^{i})\}$ denotes the set of directed edges, and *a*(*x*, *y*) denotes a directed edge from *x* to *y*. For example, in the following application study, the experiment identifying the genes whose expression is affected by smoking-induced DNA methylation: *S* indicates smoking status, *M* indicates methylation level, *Y* indicates gene expression and *g* is the number of candidate genes in the genome-wide experiment. The mechanism for each gene is depicted by a causal graph with three directed edges: smoking status points to methylation level of one site, methylation level points to gene expression, and smoking status points to gene expression; the aim is to test all the candidate mechanisms in the experiment and identify those with mediation effects. We thus propose two models given a causal graph $G^{i}$:

$$\begin{array}{c} M^{i}=\alpha_{S}^{i}S+{\left[\alpha_{X}^{i}\right]}^{T}X+\epsilon_{m},\;\epsilon_{m}∼N(0,\sigma_{m}^{2})\\ Y^{i}=\beta_{S}^{i}S+{\left[\beta_{M}^{i}\right]}^{T}M^{i}+{\left[\beta_{X}^{i}\right]}^{T}X+\epsilon_{y},\;\epsilon_{y}∼N(0,\sigma_{y}^{2}). \end{array}$$

We let ℰ={E} denote an experiment of *n* individuals with the same graphical structure (i.e. the cardinality of ℰ=n).

### 2.2 Composite null hypothesis

We omit superscript *i* for simplicity and consider the above single-mediator model, for any G in E, *α_S_* indicates the effect from *S* to *M* and *β_M_* the effect from *M* to *Y* given *S*; it can be shown that the mediation effect exists only when both *α_S_* and *β_M_* are non-zero. In other words, the null hypothesis for G is $H_{0}:\alpha_{S}=0\cup \beta_{M}=0$. Studies (Barfield *et al.* 2017, Huang 2019a) have described two important issues in this scenario. First, $H_{0}:\alpha_{S}=0\cup \beta_{M}=0$ is a *composite null* of three simple nulls that are mutually exclusive, $H_{0}=H_{0\emptyset }\cup H_{0\alpha }\cup H_{0\beta }$:

$$\begin{array}{c} H_{0\emptyset }:\alpha_{S}=0,\beta_{M}=0,\;H_{0\alpha }:\alpha_{S}\neq 0,\beta_{M}=0,\;H_{0\beta }:\alpha_{S}=0,\beta_{M}\neq 0. \end{array}$$

Ideally, the *P*-values for mediation effects should be calculated from the composite distribution consisting of the three simple nulls in a specific combination. However, the proportions for each null depend on the experiment environment and are therefore inaccessible in practice, so the exact form of the null distribution remains unknown and deriving the null distribution of any test statistics is challenging. Moreover, the re-expression of the null hypothesis, $H_{0}:\alpha_{S}\beta_{M}=0$, reveals that the test statistics consist of the product of the coefficient estimators ${\hat{\alpha }}_{S}{\hat{\beta }}_{M}$, and ${\hat{\alpha }}_{S}{\hat{\beta }}_{M}$ follows a normal product distribution. Therefore, the composite null distribution is a mixture of three different normal product distributions. Since the proportions are unknown, one may suggest using a normal approximation to resemble the underlying null distribution. However, if $H_{0\emptyset }$ is the majority of *H*_0_, the density function of ${\hat{\alpha }}_{S}{\hat{\beta }}_{M}$ under $H_{0\emptyset }$ goes to infinity at zero (Supplementary Fig. S1a). In other words, a large portion of the samples are centered at zero and a conservative conclusion is reached when a normal approximation is used to obtain *P*-values (Supplementary Fig. S1b).

### 2.3 The *P*-value of mediation effect

Without the knowledge of the proportions of three simple nulls, we can either approximate the composite null distribution or bypass the normal product statistics by using an intersection-union test. First, we construct two test statistics representing the *S—M* effect and the *M—Y* effect given *S*. Let ${\hat{\alpha }}_{S}$ and ${\hat{\beta }}_{M}$ be $\sqrt{n}$-consistent estimators for *α_S_* and *β_M_*, respectively, where $\sqrt{n}({\hat{\alpha }}_{S}-\alpha_{S})\;\overset{d}{\to }\;N(0,\sigma_{\alpha }^{2})$ and $\sqrt{n}({\hat{\beta }}_{M}-\beta_{M})\;\overset{d}{\to }\;N(0,\sigma_{\beta }^{2})$ as the sample size n→∞. We construct test statistics $a(\alpha_{S})=\sqrt{n}({\hat{\alpha }}_{S}-\alpha_{S})/(\sqrt{n}{\hat{\sigma }}_{\alpha n})$ and $b(\beta_{M})=\sqrt{n}({\hat{\beta }}_{M}-\beta_{M})/(\sqrt{n}{\hat{\sigma }}_{\beta n})$, where $n{\hat{\sigma }}_{\alpha n}^{2}\;\overset{p}{\to }\;\sigma_{\alpha }^{2}$ and $n{\hat{\sigma }}_{\beta n}^{2}\;\overset{p}{\to }\;\sigma_{\beta }^{2}$. Subsequently,

(1) $$\begin{array}{c} a\equiv a(0)\;\overset{d}{\to }\;N(0,1)\;\text{under}\;\alpha_{S}=0,\\ b\equiv b(0)\;\overset{d}{\to }\;N(0,1)\;\text{under}\;\beta_{M}=0, \end{array}$$

where *a* and *b* are independent under the *no unmeasured confounding assumptions* (details are found in Supplementary Materials); $\overset{p}{\to }$ and $\overset{d}{\to }$ denote convergences in probability and convergence in distribution, respectively.

Using *a* and *b*, we introduce three tests for assessing mediation effects. The *normality-based test* (*Sobel’s test*; Sobel 1982) approximates the composite null distribution by a normal distribution:

(2) $$p_{N}(a,b)=2\times \Phi \left(\frac{-|ab|}{\sqrt{a^{2}+b^{2}}}\right),$$

where Φ(·) is the cumulative distribution function of standard normal. The *joint significance test* (MacKinnon *et al.* 2002), an intersection-union test that avoids normal product statistics:

(3) $$p_{\text{JS}}(a,b)=2\times \max\{\Phi (-|a|),\Phi (-|b|)\},$$

And *the composite test* proposed by Huang (2019a) provides *P*-values without estimating the unknown proportion of three simple nulls in *H*_0_:

(4) $$p_{\text{comp}}(a,b)=F(\frac{ab}{\sqrt{\text{Var}(a)}})+F(\frac{ab}{\sqrt{\text{Var}(b)}})-F(ab)+\epsilon ,$$

where *F*(*z*) is the probability density function of the normal product distribution and can be calculated through numerical integration, *ϵ* is bounded and can be ignored when $H_{0\emptyset }$ is dominant in *H*_0_ (check the details in Huang 2019a). The variances of *a* and *b* depend on the proportion of the three simple nulls and hence can only be estimated on a collection of hypothesis tests for a similar purpose, e.g. a series of tests in a genome-wide analysis. Thus, the *P*-values derived from the composite test can be construed as we approximate the composite null distribution by a data-driven proportion, and by using that proportion, we can ensure the genome-wide *P*-values are also uniformly distributed under the composite null.

### 2.4 Multimediator model

**Figure 1.**
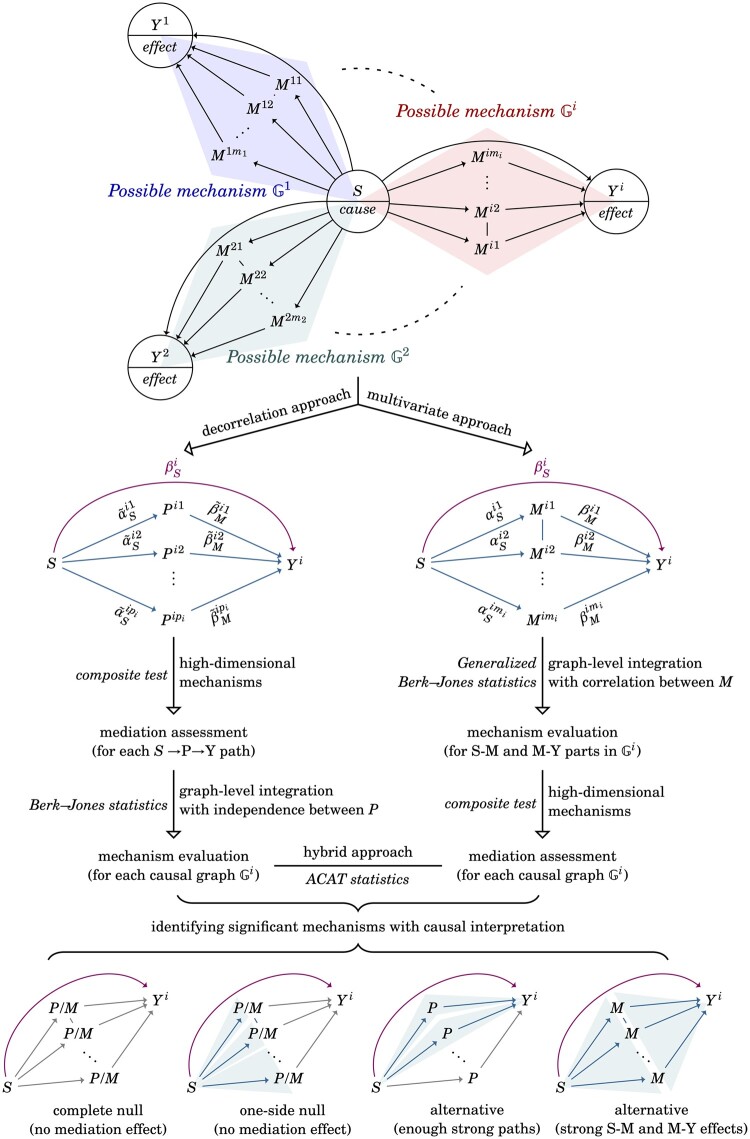
Method overview. An experiment involving *g* causal graphs. The *i*-th causal graph $G^{i}$ is constructed by the exposure *S*, vector of mediators $M^{i}$, and outcome *Y^i^*, with a total of *n* individuals and *m_i_* mediators (or corresponding *p_i_* decorrelated mediators). Using the following application as an example, this experiment aims to identify the genes whose expression is affected by smoking-induced DNA methylation: *S* indicates smoking status, $M^{i}$ indicates the expression level of multiple methylation sites on the *i*-th gene, *Y^i^* indicates gene expression of the *i*-th gene and *g* is the number of candidate genes in the genome-wide experiment. The emerald paths indicate the mediation effect, i.e. the change in gene expression regulated by smoking-induced DNA methylation, and the purple path indicates a direct effect that is not mediated by the mediators, i.e. the change in gene expression regulated by smoking but not through the DNA methylation. Four possible results of mediation assessment are also illustrated; colored paths and background indicate regions of non-zero signals.

We generalize the single-mediator model into the multimediator model as depicted in Fig. 1. The *i*-th causal graph is defined as a mixed graph $G^{i}=\{V^{i},E^{i},A^{i}\}$, where $V^{i}=\{S,M^{i},Y^{i}\}$ denotes the set of vertices, $M^{i}=(M^{ij})$ denotes the vector of mediators, $j\in \{1,\ldots ,m_{i}\},\;E^{i}=\{e(M^{ij},M^{ik})|j\neq k\}$ denotes the set of undirected edges, *e*(*x*, *y*) denotes an undirected edge between *x* and *y*, $A^{i}=\{a(S,M^{ij}),a(M^{ij},Y^{i}),a(S,Y^{i})\}$ denotes the set of directed edges. The causal diagram illustrates a scenario in which (i) the mediators are correlated within a causal graph but independent between the graphs (i.e. each possible mechanism should be regarded as a self-contained unit), and (ii) the causal relationships between the exposure, mediators, and outcome are predefined, but the causal directions between the mediators remain ambiguous such that some flexibility can be preserved. Using the example in the following application study, the causal graph assumes multiple regulating paths from smoking to several methylation sites to corresponding gene expression, and these paths may influence each other; however, we assume the regulating paths across different graphs are independent since these paths direct to different genes. We thus propose two models given a causal graph $G^{i}$:

(5) $$M^{i}=\alpha_{S}^{i}S+{\left[\alpha_{X}^{i}\right]}^{T}X+\epsilon_{m},\;\epsilon_{m}∼\text{MVN}(0,\Sigma_{m}),$$

(6) $$Y^{i}=\beta_{S}^{i}S+{\left[\beta_{M}^{i}\right]}^{T}M^{i}+{\left[\beta_{X}^{i}\right]}^{T}X+\epsilon_{y},\;\epsilon_{y}∼N(0,\sigma_{y}^{2})$$

where $\alpha_{S}^{i}=(\alpha_{S}^{ij}),\;\alpha_{X}^{i}=(\alpha_{X}^{i1},\ldots ,\alpha_{X}^{im_{i}}),\;\beta_{M}^{i}=(\beta_{M}^{ij}),\;\alpha_{X}^{ij}$ and $\beta_{X}^{i}$ are vectors with a length equal to the number of covariates. We can formulate the null hypothesis for testing $G^{i}$ as follows:

(7) $$H_{0}:\alpha_{S}^{i}=0\cup \beta_{M}^{i}=0.$$

The composite null hypothesis becomes complicated due to multiple correlated mediators; for example, one may consider *disjoint effect* ($\alpha_{S}^{1}=0,\beta_{M}^{1}\neq 0,\alpha_{S}^{2}\neq 0,\beta_{M}^{2}=0$) or *perfect cancellation* ($\alpha_{S}^{1}\beta_{M}^{1}\neq 0,\alpha_{S}^{2}\beta_{M}^{2}\neq 0$ but $\alpha_{S}^{1}\beta_{M}^{1}+\alpha_{S}^{2}\beta_{M}^{2}=0$). Therefore, further model assumptions of *no disjoint effects as null* and *no perfect cancellation* are required. Both assumptions are acceptable in practice since exact independence or exact cancellation only happens with zero probability (Weinberger 2018; details are found in Supplementary Materials).

The main difference between the single-mediator and the multimediator models lies in the dependency between estimators in $G^{i}$ (i.e. in a multimediator model, α^Sip⊥⊥α^Siq and β^Mip⊥⊥β^Miq for arbitrary *p* and *q* due to the existence of correlated mediators). The independence among estimators ${\hat{\alpha }}_{S}^{i}$ and among estimators ${\hat{\beta }}_{M}^{i}$ in $G^{i}$ are required to apply the composite test. Therefore, we propose a *decorrelation approach* and a *multivariate approach* for achieving the required independence.

#### 2.4.1 Decorrelation approach: use BJ with the composite test

The main steps of the decorrelation approach include (i) applying decorrelation between mediators, (ii) using path-level statistics to assess the mediation effect under the composite null, and (iii) integrating path-level statistics into one graph-level statistics for $G^{i}$.

##### 2.4.1.1 Step 1—decorrelation between mediators

The vector of *decorrelated mediators*, $P^{i}=(P^{ij})$, is a linear combination of original mediators whose residuals have an orthogonal covariance matrix given the exposure and covariates, $P^{i}=u^{i}M^{i}$. Here, we obtain **u** using the eigendecomposition of ${\hat{\epsilon }}_{m}$, i.e. the estimate of $\epsilon_{m}$ in Equation (5). Thus, the regression model in Equations (5) and (6) becomes

(8) $$P^{i}={\tilde{\alpha }}_{S}^{i}S+{\left[{\tilde{\alpha }}_{X}^{i}\right]}^{T}X+\epsilon_{p},\;\epsilon_{p}∼\text{MVN}(0,D),$$

(9) $$Y^{i}=\beta_{S}^{i}S+{\left[{\tilde{\beta }}_{M}^{i}\right]}^{T}P^{i}+{\left[\beta_{X}^{i}\right]}^{T}X+\epsilon_{y},\;\epsilon_{y}∼N(0,\sigma_{y}^{2}),$$

where **D** is a diagonal matrix, and the correlation between ${\tilde{\alpha }}_{S}^{i}$ and between ${\tilde{\beta }}_{M}^{i}$ are also eliminated due to the decorrelation transformation from $M^{i}$ to $P^{i}$. The tilde coefficients are also the linear combination of the original coefficients. The decorrelation step can reduce the dimension of mediators (i.e. eliminate the dimensions of small eigenvalues), preserve the truth value of the null hypothesis, and identify disjoint effects as mediation effects. A demonstrative example is found in Supplementary Materials.

##### 2.4.1.2 Step 2—mediation assessment under a composite null

The tilde coefficients for each $G^{i}$ estimated in step 1 must be transformed into a Gaussian distribution to satisfy the requirement for applying Equation (4). Using Equation (1), we transform each ${\hat{\alpha }}_{S}^{ij}$ into *a^ij^*, transform ${\hat{\beta }}_{M}^{ij}$ into *b^ij^*, and concatenate them into a vector of experiment level, namely $a_{\sum p_{i}\times 1}={\left({\left[a^{1}\right]}^{T},\ldots ,{\left[a^{g}\right]}^{T}\right)}^{T}=(a^{k})$ and $b_{\sum p_{i}\times 1}={\left({\left[b^{1}\right]}^{T},\ldots ,{\left[b^{g}\right]}^{T}\right)}^{T}=(b^{k})$, where $a^{i}=(a^{ij}),\;b^{i}=(b^{ij})$, and $k\in \{1,\ldots ,\sum_{i=1}^{g}p_{i}\}$. Here, the original *ij*-index indicates the *j*-th path in the *i*-th graph, and the *k*-index indicates the *k*-th path in the genome-wide experiment. Next, we can obtain a *P*-value for each path ($S\to P^{ij}\to Y^{i}$) in the experiment. For $k\in \{1,\ldots ,\sum p_{i}\}$, the *P*-value of the *k*-th path can be calculated using Equations (2–4):

(10) $${\hat{p}}_{N}^{k}={\hat{p}}_{N}(a^{k},b^{k}),\;{\hat{p}}_{\text{JS}}^{k}={\hat{p}}_{\text{JS}}(a^{k},b^{k}),\;{\hat{p}}_{\text{comp}}^{k}={\hat{p}}_{\text{comp}}(a^{k},b^{k}).$$

Finally, we obtain the set of path-level *P*-values of mediation effects, $\left\{{\hat{p}}^{ij}\}=\{{\hat{p}}^{k}\right\}$, where ${\hat{p}}^{k}$ can be ${\hat{p}}_{N}^{k},\;{\hat{p}}_{\text{JS}}^{k}$, or ${\hat{p}}_{\text{comp}}^{k},\;k=\sum_{u=0}^{i-1}p_{u}+j$ and $p_{0}=0$. Then we transform them into *Z* statistics using the cumulative distribution function of a standard normal distribution

(11) $$\Phi^{-1}(1-p/2)=Z,$$

where $\Phi^{-1}(\cdot )$ denotes the inverse of Φ(·) and $\bar{\Phi }(\cdot )=1-\Phi (\cdot )$. Here, *p* can be any legit *P*-value, so either ${\hat{p}}_{N}^{k},\;{\hat{p}}_{\text{JS}}^{k}$, or ${\hat{p}}_{\text{comp}}^{k}$ is qualified. We then obtain $\left\{Z^{ij}\right\}$ for each $G^{i}$ and denote $Z_{N}^{i},\;Z_{\text{JS}}^{i}$, and $Z_{\text{comp}}^{i}$ as the vectors of *Z*-statistics derived from $\left\{{\hat{p}}_{N}^{ij}\},\;\{{\hat{p}}_{\text{JS}}^{ij}\right\}$, and $\left\{{\hat{p}}_{\text{comp}}^{ij}\right\}$, respectively.

##### 2.4.1.3 Step 3—integration into graph-level statistics

**Figure 2.**
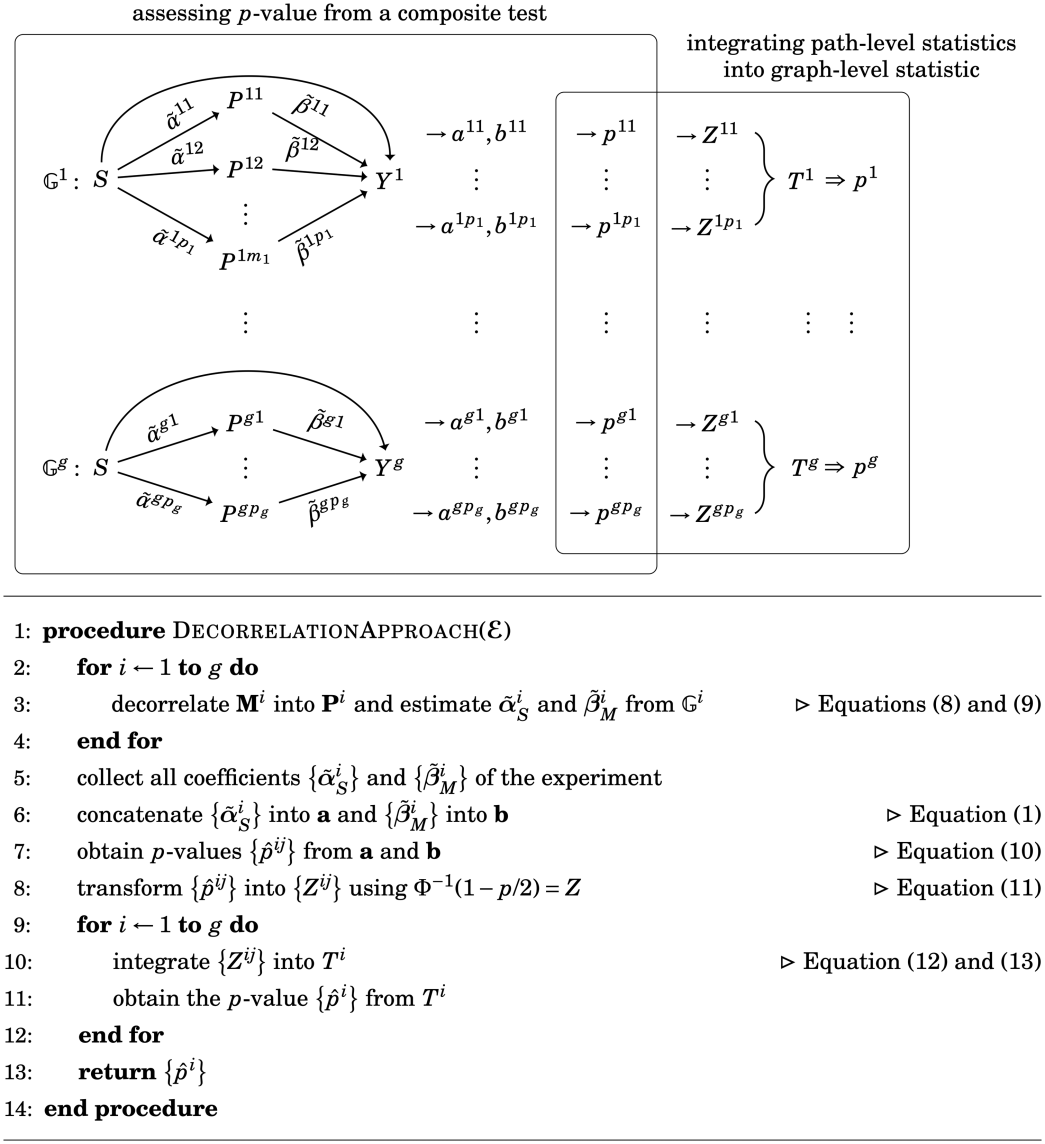
Decorrelated approach. The decorrelation approach consists of three main steps: (a) decorrelation of mediators as described in Equations (8) and (9), (b) mediation assessment using a composite test, as described in Equation (10), and (c) integration of the path-level *P*-values into a graph-level statistic for $G^{i}$.

After collecting the *Z*-statistics for all paths in the experiment, the next step is to evaluate each graph by a BJ statistics (Berk and Jones 1979). We ignore the superscript *i* (i.e. the graph-level index) since *T^i^* only considers the information within one graph. Here, a random variable *S* is defined to indicate the number of significant paths in the graph:

(12) $$S(t)=\sum_{j=1}^{m}I\;(|Z^{j}|\geq t)∼\text{Binomial}\{m,2\bar{\Phi }(t)\},\;\text{for}\;\text{any}\;\text{fixed}\;t\geq 0,$$

where I(·) denotes an indicator function. Then, the *BJ* statistic integrating the independent paths in one causal graph is thus:

(13) $$\begin{array}{c} T_{\text{BJ}}(Z)=\underset{1\leq j\leq m/2}{\max}\;\text{log}\;\left[\frac{\text{Pr}\{S(|Z{|}^{\left(m-j+1\right)})=j|E(Z)={\hat{\mu }}_{j,m}\cdot 1\}}{\text{Pr}\{S(|Z{|}^{\left(m-j+1\right)})=j|E(Z)=\mu_{0}\}}\right]\cdot I\left\{2\bar{\Phi }(|Z{|}^{\left(m-j+1\right)})<\frac{j}{m}\right\}, \end{array}$$

where $|Z{|}^{\left(1\right)},\ldots ,|Z{|}^{\left(m\right)}$ denote the order statistics from the absolute value of **Z**, $\left|Z^{1}|,\ldots ,|Z^{m}\right|$, so $|Z{|}^{\left(1\right)}$ is of the smallest value of **Z** in magnitude, $\mu_{0}={\left(0,0,\ldots ,0\right)}_{1\times m},\;1={\left(1,1,\ldots ,1\right)}_{1\times m}$, and ${\hat{\mu }}_{j,m}>0$ can be estimated from the solution to the equation $j/m=1-\{\Phi (|Z{|}^{\left(m-j+1\right)}-{\hat{\mu }}_{j,m})-\Phi (-|Z{|}^{\left(m-j+1\right)}-{\hat{\mu }}_{j,m})\}$. We use the *P*-values of BJ statistics, namely $T_{\text{BJ}}(Z_{N}^{i}),\;T_{\text{BJ}}(Z_{\text{JS}}^{i})$, and $T_{\text{BJ}}(Z_{\text{comp}}^{i})$, to evaluate mechanism $G^{i}$. The *P*-values of the BJ statistics can be calculated using the method provided by Moscovich and Nadler (2017). Refer to Fig. 2 for a detailed illustration and the algorithm.

#### 2.4.2 Multivariate approach: use GBJ with the composite test

In our model settings, mediators are correlated within a mechanism but are independent across mechanisms. In other words, the *independence at mechanism level* as described in Supplementary Fig. S5 can be used to fulfill the requirement of using Equation (4). Therefore, the core concept of the multivariate approach is to integrate the information from *S* to **M** into one statistic $T_{\alpha }$; from **M** to *Y* into another statistic $T_{\beta }$, and then use Equation (16) to obtain the *P*-value of mediation effect.

##### 2.4.2.1 Step 1—integration into graph-level statistics

The first step is to provide graph-level statistics that integrate all paths and their dependency in causal graphs to achieve independence at the graph level. Two test statistics for $G^{i}$, namely $T_{\alpha }^{i}$ and $T_{\beta }^{i}$, are constructed with the correlated estimators ${\hat{\alpha }}_{S}^{i}$ and ${\hat{\beta }}_{M}^{i}$ and their covariance matrices. Hence, $T_{\alpha }^{i}$ indicates the strength for the paths $S\to M^{i}$ and $T_{\beta }^{i}$ for $M^{i}\to Y^{i}$ given *S*, and their product represents the causal graph $S\to M^{i}\to Y^{i}$. The superscript *i* is ignored from here. Let $Z_{\alpha }$ and $Z_{\beta }$ be the statistics rescaling the variance of ${\hat{\alpha }}_{S}$ and ${\hat{\beta }}_{M}$ to 1, and let their correlation matrices be $\Sigma_{\alpha }$ and $\Sigma_{\beta }$, respectively. The BJ statistic is then extended to incorporate a correlation structure, namely the *GBJ* statistic,

(14) $$\begin{array}{c} T_{\text{GBJ}}(Z,\Sigma )=\underset{1\leq j\leq m/2}{\max}\;\text{log}\;\left[\frac{\text{Pr}\{S(|Z{|}^{\left(m-j+1\right)})=j|E(Z)={\hat{\mu }}_{j,d}\cdot 1,\text{cov}(Z)=\Sigma \}}{\text{Pr}\{S(|Z{|}^{\left(m-j+1\right)})=j|E(Z)=\mu_{0},\text{cov}(Z)=\Sigma \}}\right]\cdot I\left\{2\bar{\Phi }(|Z{|}^{\left(m-j+1\right)})<\frac{j}{m}\right\}, \end{array}$$

and we obtain $T_{\alpha }^{i}=T_{\text{GBJ}}(Z_{\alpha }^{i},\Sigma_{\alpha }^{i})$ and $T_{\beta }^{i}=T_{\text{GBJ}}(Z_{\beta }^{i},\Sigma_{\beta }^{i})$ for each $G^{i}$. The *P*-values of the GBJ statistics can be calculated using a generalized version of Moscovich-Eiger and Nadler’s method, provided by Sun and Lin (2020).

##### 2.4.2.2 Step 2—mediation assessment under a composite null

We transform $T_{\alpha }^{i}$ and $T_{\beta }^{i}$ into normally distributed random variables *a^i^* and *b^i^*, respectively, to apply Equation (4):

(15) $$a^{i}=\text{sign}({\hat{\alpha }}_{S}^{i})\Phi^{-1}(1-p_{\alpha }^{i}/2),\;b^{i}=\text{sign}({\hat{\beta }}_{M}^{i})\Phi^{-1}(1-p_{\beta }^{i}/2),$$

where $p_{\alpha }^{i}$ and $p_{\beta }^{i}$ are the *P*-values of $T_{\alpha }^{i}$ and $T_{\beta }^{i}$, respectively, and sign$\left({\hat{\theta }}^{i})=\{1-2I(\sum_{j=1}^{m_{i}}{\hat{\theta }}^{ij}<0)\right\}$ is a function for determining the collective direction of regression coefficients (i.e. sign$(\hat{\theta })=1$ if the summation of the direction is positive; otherwise, sign$(\hat{\theta })=-1$). Similar to Equation (1), we prove that *a^i^* and *b^i^* also converge to standard normal distribution under the null; the proof is found in Supplementary Material. We then concatenate *a^i^* and *b^i^* into a vector of experiment level, namely $a_{1\times g}={\left(a^{1}\ldots ,a^{g}\right)}^{T}$ and $b_{1\times g}={\left(b^{1},\ldots ,b^{g}\right)}^{T}$, and obtain a *P*-value for each causal graph from *S* toward *Y^i^* in the experiment by using Equations (2–4):

(16) $${\hat{p}}_{N}^{i}={\hat{p}}_{N}(a^{i},b^{i}),\;{\hat{p}}_{\text{JS}}^{i}={\hat{p}}_{\text{JS}}(a^{i},b^{i}),\;{\hat{p}}_{\text{comp}}^{i}={\hat{p}}_{\text{comp}}(a^{i},b^{i})$$

**Figure 3.**
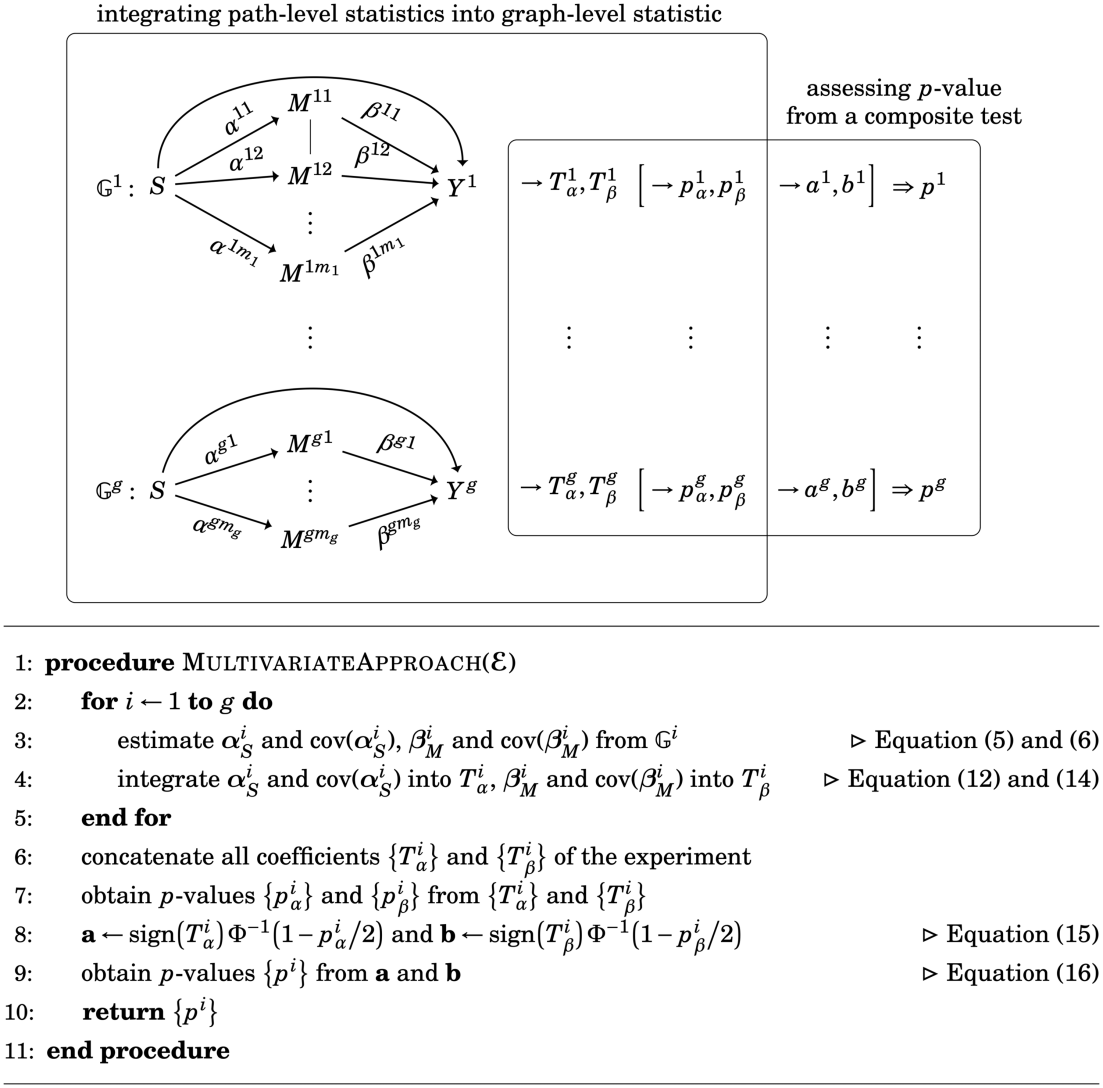
Multivariate approach. The multivariate approach consists of two main steps: (a) the integration of path coefficients into two multivariate statistics for $G^{i}$ and (b) mediation assessment using a composite test as described in Equation (16).

We use the *P*-values of mediation effect, namely ${\hat{p}}_{N}^{i},\;{\hat{p}}_{\text{JS}}^{i}$, and ${\hat{p}}_{\text{comp}}^{i}$, to evaluate mechanism $G^{i}$. Refer to Fig. 3 for a detailed illustration and the algorithm.

#### 2.4.3 Hybrid approach: use ACAT to integrate BJ and GBJ

In the following simulation, we demonstrate that both approaches have their edges. The decorrelation approach prefers effects with a consistent direction (e.g. most of the relevant pathways are upregulated or downregulated), but the multivariate prefers effects with disturbance (e.g. some pathways are upregulated but some are downregulated). For those who have limited knowledge of their data, we also provide a hybrid approach that integrates both approaches without raising the burden of multiple comparisons. The aggregated Cauchy association test (ACAT) (Liu and Xie 2020) statistic is able to integrate correlated *P*-value without estimating their correlation. Here, the *i*-th ACAT statistics for the mediation effect can be defined as

(17) $$T_{\text{ACAT}}^{i}(p_{\text{decor}}^{i},p_{\text{multi}}^{i})=w_{\text{decor}}\;\text{tan}[\{0.5-p_{\text{decor}}^{i}\}\pi ]+w_{\text{multi}}\;\text{tan}[\{0.5-p_{\text{multi}}^{i}\}\pi ],$$

where $w_{\text{decor}}$ and $w_{\text{multi}}$ are arbitrary weights that can be incorporated as prior knowledge, and the component tan[{0.5−p)}π] follows a standard Cauchy distribution under the null. The *P*-value of ACAT is approximated by $p_{\text{ACAT}}\approx 1/2-\arctan\{T_{\text{ACAT}}/(w_{\text{decor}}+w_{\text{multi}})\}/\pi$.

We conclude the method section by highlighting the thinking behind the proposed approaches. Huang (2019a) proved that, in a genome-wide analysis, the composite test outperforms the joint significance test and the normality-based test when only a few mechanisms are causally involved, but the study was limited to single-mediator models. We generalize the method into multimediator models of correlated mediators, and we provide two approaches to achieve the independence between the test statistics required in the composite test. The decorrelation approach achieves independence at the path level by directly decorrelating the mediators; the multivariate approach uses GBJ statistics to incorporate correlation between estimators and achieves independence at the mechanism level. We also prove that the test statistics in the multivariate approach converge to the standard normal distributions (i.e. the test statistics behave in the same way as they do in the single-mediator model in Huang (2019a), the proof is found in Supplementary Material). As an alternative option, a hybrid approach using ACAT to capture the properties of both approaches is also provided.

## 3 Results and discussion

### 3.1 Simulations

We performed comprehensive simulation analyses to demonstrate the strengths and limitations of the decorrelation approach and multivariate approach. The hybrid approach integrates two *P*-values using a ready-made statistic; therefore, as long as the decorrelation and multivariate approaches are able to control type I error, the hybrid results also follow. Here, we only focus on the performance of the hybrid approach under the alternative. The proposed approaches can be applied to other *P*-value integration statistics or multivariate statistics as well, so we included higher criticism (HC) (Donoho and Jin 2004) and the minimum *P*-value test (minP; Conneely and Boehnke 2007) in the decorrelation approach, generalized HC (GHC) (Barnett *et al.* 2017), minP, Hotelling’s *T*-squared (TSQ) statistic (Hotelling 1931), and the variance component test (VCT) (Huang 2019b) in the multivariate approach for comparison. The general setting for one genome-wide experiment included 1000 individuals and 10 000 mechanisms, where 10% of the 10 000 mechanisms (i.e. pathway from treatment toward gene or protein) included mediation effects. For the *i*-th mechanism, we first randomly generated the exposure *S* and two covariates **X** by using Normal(0, 1); we then generated 30 potential mediators by Equation (5), where $\alpha_{X}^{i}=1$ and $\Sigma^{i}$ is a correlation matrix with an off-diagonal equal to the correlation coefficient ρ=0.3, which is the setting of Sun and Lin (2020) to ensure the matrix is positive definite. Finally, we generated the outcome *Y* by Equation (6), where $\beta_{S}^{i}=1$ and $\beta_{X}^{i}=1$. For each mechanism with a non-zero mediation effect, we assigned *h* mediators with a signal from the 30 *M*’s; the nonzero signals *α_S_* and *β_M_* of the *h* mediators followed Normal(μ,v), and the remaining (30−h)M without signals had coefficients of zero. We modified selected criteria in the general settings to evaluate different scenarios, as presented in Supplementary Table S1.

### 3.2 Performance under the null

We demonstrated that the proposed approaches are sufficiently accurate for controlling the genome-wide type I error rate. We simulated three types of null scenarios, as described in Supplementary Table S1a, and the detailed results are summarized in Supplementary Table S2. First, we compared three *P*-values of mediation effects introduced in Equations (2–4). Supplementary Figure S6 shows that only the significant proportions of the composite test reach the predetermined *α* level in this genome-wide simulation; the composite null behind the mediator model led to conservative results in both normality-based and joint significant tests, and this observation is consistent with the previous results by Huang (2019a). Then, we focused on the test statistics with the composite test. As shown in Table 1, the composite test produced uniformly distributed *P*-values, so we are able to control the type I error up to the significance level $\alpha =10^{-5}$ using both the decorrelation and multivariate approaches. Notice that the multivariate approach may induce inflated type I error rates in the case of correlated data under the one-sided null. We found that GHC is less tolerable to the correlated data, and both GHC and minP are inflated under the one-sided null. Additionally, TSQ can be seriously inflated due to insufficient sample size (blue cells in Supplementary Table S2), so we leave its results with detailed simulations and discussion in Supplementary Materials. The disjoint case with correlated mediators is a scenario under the alternative for both approaches (red cells in Supplementary Table S2), and the disjoint null with independent mediators is excluded by in our assumption (*no disjoint effects as null*, discussion and proof are found in Supplementary Material), so we leave their results in Supplementary Materials.

**Table 1.** Type I error using the composite test.^a^

Nominal *α* level	Hypothesis	Mediator setting	Decorrelation approach			Multivariate approach			
			BJ	HC	minP	VCT	GBJ	GHC	minP
0.01	Complete	independent	0.0100	0.0103	0.0103	0.0097	0.0075	0.0102	0.0099
		correlated	0.0101	0.0104	0.0104	0.0098	0.0073	0.0108	0.0099
	One-sided	independent	0.0102	0.0104	0.0105	0.0097	0.0078	0.0103	0.0102
		correlated	0.0099	0.0103	0.0102	0.0099	0.0077	0.0111	0.0101
0.001	Complete	independent	0.0010	0.0011	0.0011	0.0009	0.0006	0.0011	0.0010
		correlated	0.0011	0.0011	0.0011	0.0009	0.0007	0.0012	0.0010
	One-sided	independent	0.0010	0.0011	0.0011	0.0010	0.0008	0.0013	0.0013
		correlated	0.0010	0.0011	0.0011	0.0011	0.0008	0.0013	0.0011
1e−04	Complete	independent	1.08e−04	1.18e−04	1.18e−04	8.40e−05	6.30e−05	9.70e−05	9.00e−05
		correlated	1.10e−04	1.11e−04	1.11e−04	9.10e−05	8.70e−05	**1.42e−04**	1.08e−04
	One-sided	independent	1.17e−04	1.19e−04	1.19e−04	9.30e−05	1.13e−04	**2.31e−04**	**2.29e−04**
		correlated	1.00e−04	1.12e−04	1.12e−04	**1.44e−04**	**1.47e−04**	**2.40e−04**	**2.03e−04**
1e−05	Complete	independent	1.10e−05	1.40e−05	1.40e−05	8.00e−06	5.50e−06	1.15e−05	9.00e−06
		correlated	1.27e−05	1.27e−05	1.27e−05	1.13e−05	1.23e−05	**1.73e−05**	1.13e−05
	One-sided	independent	7.50e−06	1.20e−05	1.20e−05	**1.40e−05**	**2.20e−05**	**5.45e−05**	**5.20e−05**
		correlated	9.00e−06	1.07e−05	1.07e−05	**2.33e−05**	**2.83e−05**	**5.37e−05**	**4.50e−05**

a Generally, we control the type I error to the significance level $\alpha =10^{-5}$ using both approaches. The multivariate approach may induce inflated type I error rates (highlighted in boldface) in the case of correlated mediators under the one-sided null: GHC is less tolerable to correlated data, and both GHC and minP are inflated under one-side nulls.

### 3.3 Performance under the alternative

**Figure 4.**
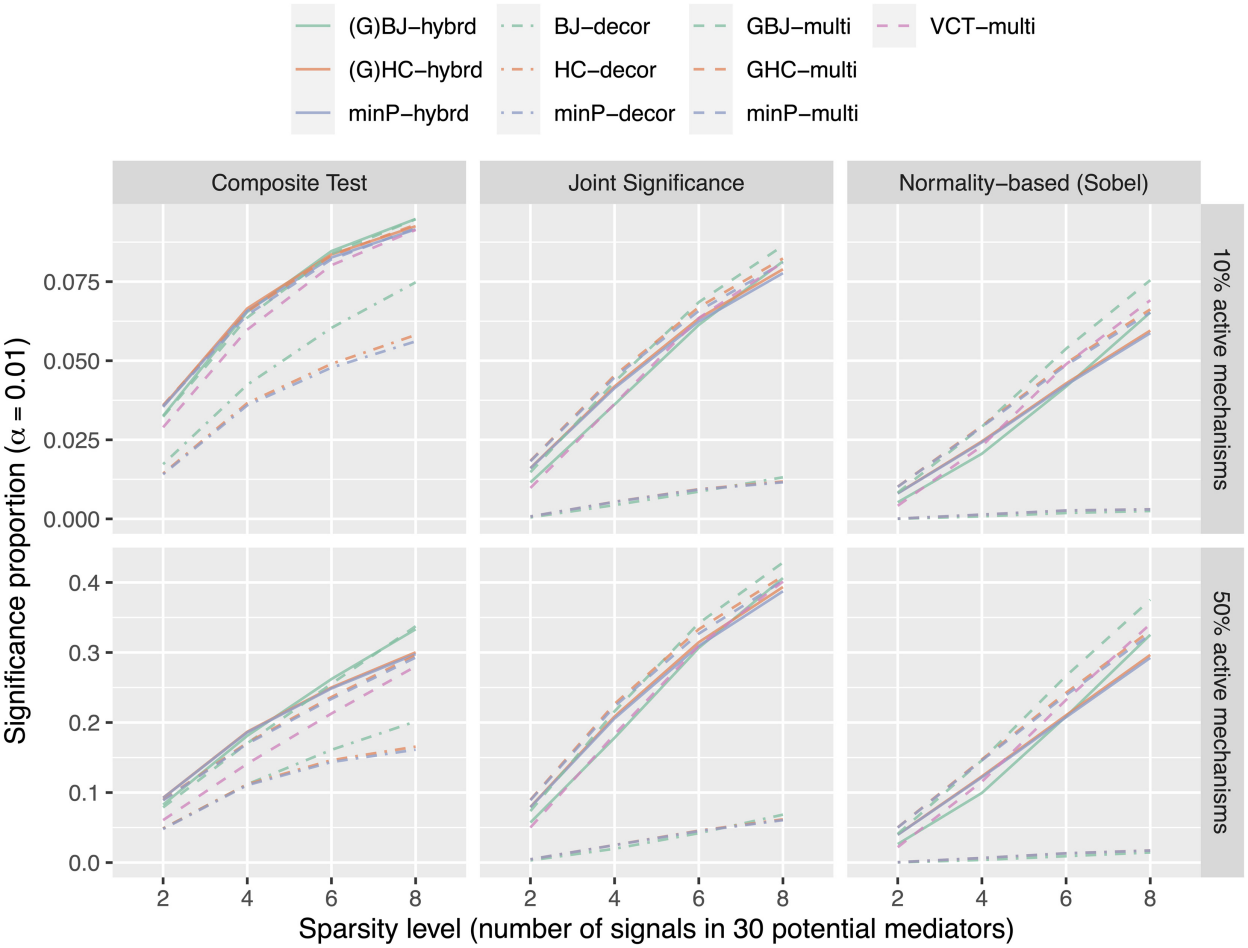
The performance of the proposed approaches in different sparsity levels. We compared the two proposed approaches with three assessments of the mediation effect. We let 10% and 50% of mechanisms have signals (i.e. 1000 and 5000 of the 10 000 mechanisms in one experiment, respectively), the signal mean was fixed at 0.05 with a random variance setting (details in Supplementary Table S1). The significance level was 0.01. The composite test outperformed the other tests for the dataset in which 10% of the mechanisms had signals, and the power of the joint significance test increased for the dataset in which 50% of the mechanisms had signals.

We performed comprehensive simulations as described in Supplementary Table S1b. We first demonstrated the performance of the composite test in a genome-wide simulation of sparse signals. We considered two levels of sparsity: the mechanism-level sparsity, i.e. the number of active S→M→Y paths within a mechanism G, and the experiment-level sparsity, i.e. the number of active mechanisms within an experiment. Figure 4 shows that the composite test is much more powerful than the other two tests when signals are sparse at both mechanism and experiment levels. The composite test performs better than the others in the scenario of 10% active mechanisms, and when the number of active mediators is also small, e.g. x≤4, we found the composite test had a huge advantage over other tests. We found that the multivariate approach generally performed better than the decorrelation approach, and signal detection was extremely difficult when the decorrelation approach was adopted for the joint significance test and normality-based test. When the multivariate approach was adopted, the joint significance test may more powerfully detect dense signals at the experiment level (e.g. the scenario of 50% active mechanism).

Figure 4 also shows that BJ and GBJ perform well among the decorrelation statistics and multivariate statistics, respectively. We also performed a simulation of *m *=* *100 in Supplementary Fig. S8 to focus on different sparse regions suggested by Sun and Lin (2020), and we found GBJ indeed performs better in the moderately sparse region as they suggested. Comparing the signal patterns, we found that consistent signals (i.e. similar signal mean shifts; in our setting μ=0.05,v=0) favor TSQ, VCT, and the decorrelation approaches; diverse signals (i.e. larger signal variation without obvious mean shifts in average; in our setting μ=0,v=0.1) favor the multivariate approach over the decorrelation approach. When we compared the case of mixed signals (i.e. large signal variance with obvious mean shifts; in our setting μ=0.05,v=0.1) with the other two cases, we discovered that the multivariate approaches were more sensitive to diverse signals and the decorrelation approaches were more sensitive to consistent signal.

Supplementary Figure S8 shows that the hybrid approach is able to capture the method with better performance. The hybrid approach is in favor of the multivariate approach when signals are diverse and in favor of the decorrelation approach when signals are consistent. Figure 4 and Supplementary Fig. S7 also show that incorporating the composite test with the hybrid approach has the best performance. Other discussions are found in Supplementary Materials.

We summarize the simulations section: (i) we are able to control the type I error in the proposed multimediator approaches, (ii) the composite test outperforms other tests of mediation effect in both levels of sparsity, (iii) the multivariate approach is more sensitive to diverse signals, the decorrelation approach to consistent signals, and the hybrid approach captures both edges, and (iv) GBJ is a fair choice among the multivariate statistics because its flexibility to tolerate mild correlation and one-side null (tolerable than GHC and minimum *P*-values, Table 1), robustness to smaller sample size (not inflated as soon as TSQ, Supplementary Table S2), and it only requires GWAS summary *Z*-statistics to perform (where VCT requires individual-level data). In brief, we suggest the users use the hybrid approach if the underlying mechanisms are unknown. Otherwise, one can choose either or both approaches to capture the target genes with the desired properties. The decorrelation and multivariate approaches represent different types of biological changes, and as we can see in the data application, the genes identified from both approaches only have a few overlaps.

### 3.4 Data application

We present two data applications using multiomic datasets from TCGA-LUAD (Network *et al.* 2014) and CPTAC-LUAD (Gillette *et al.* 2020). After preprocessing (details are found in Supplementary Materials), the *methyl-gene dataset* from TCGA included 20 670 genes and the information on 416 individuals with complete data regarding sex, age, race, tumor stage, and smoking records. We regarded the smoking status as the exposure *S* (61 nonsmokers versus 355 smokers), *i*-th gene expression as the outcome *Y^i^*, and methylation levels related to the target gene as the mediators $M^{i}$. The mapping of methylation sites to their target gene is provided by the GDC reference files (HM450, hg38, v22) from the NCI website. Similarly, we applied the proposed method on the *miRNA-protein dataset* from CPTAC, in which we had sex, age, race, and smoking records of 99 individuals with at most 20% missing data. The dataset included 6955 proteins regulated by miRNA; the smoking status is denoted as *S* (46 nonsmokers versus 53 smokers), *i*-th protein expression as *Y^i^*, and miRNA expression related to the target protein as $M^{i}$. The relationship between miRNAs and their targets was retrieved from the database miRTarBase (Huang *et al.* 2020; access date: 16 August 2022). This database provides miRNA–target interactions verified by manually curated articles and CLIP-seq data.

**Figure 5.**
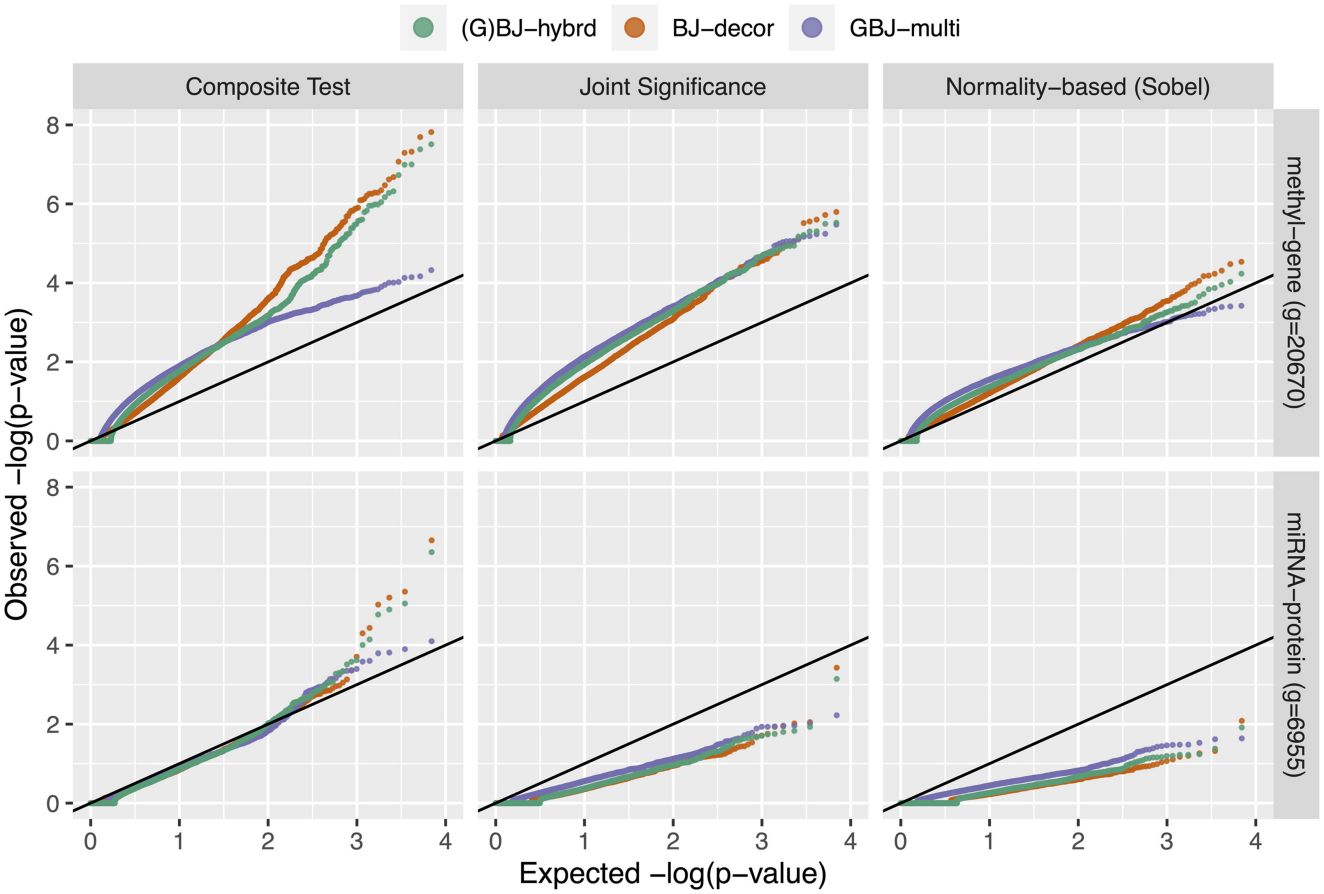
Q–Q plots obtained from the three assessments of mediation effects. We found that the methyl-gene dataset presented strong and consistent signals at a genome-wide scale, but the miRNA-protein dataset only reported a few significant proteins. Another Q–Q plot for all statistics we used in the simulation is shown in Supplementary Fig. S11. This plot excluded a few extreme *P*-values for the clarity of visualization.

We used the two datasets to demonstrate the scenarios of different experimental scales: a general genome-wide study ($∼2\times 10^{4}$ genes) and a specific proteome-wide study (a few thousand proteins, e.g. phosphoproteomic data). The Manhattan plots of the methyl-gene dataset are illustrated in Supplementary Fig. S10, and the Q–Q plots of the two datasets are illustrated in Fig. 5. We found that the miRNA-protein dataset described a very weak scenario on a proteome-wide scale; the Q–Q plot of the composite test indicated that protein expression could hardly be influenced by smoking-induced miRNA expression, and the plots of joint significance and normality-based tests are conspicuously underpowered. Although there may exist some proteins regulated by the downstream of smoking-induced miRNA expression, that is not a systematic scheme in this dataset from a genome-wide point of view. On the other hand, the methyl-gene dataset presented strong and consistent signals; all Q–Q plots indicated that gene expression could be influenced by smoking-induced methylation expression. The pattern is also supported by the Q–Q plots using all statistics in Supplementary Fig. S11. Similar to the simulation results, Supplementary Fig. S11 also shows that the joint significance and normality-based tests are underpowered when signals are weak, and the performances between (G)BJ, (G)HC, and minP statistics are comparable. Then we focused on the analyses of the methyl-gene dataset using BJ-decor, GBJ-multi, and (G)BJ-hybrid.

**Figure 6.**
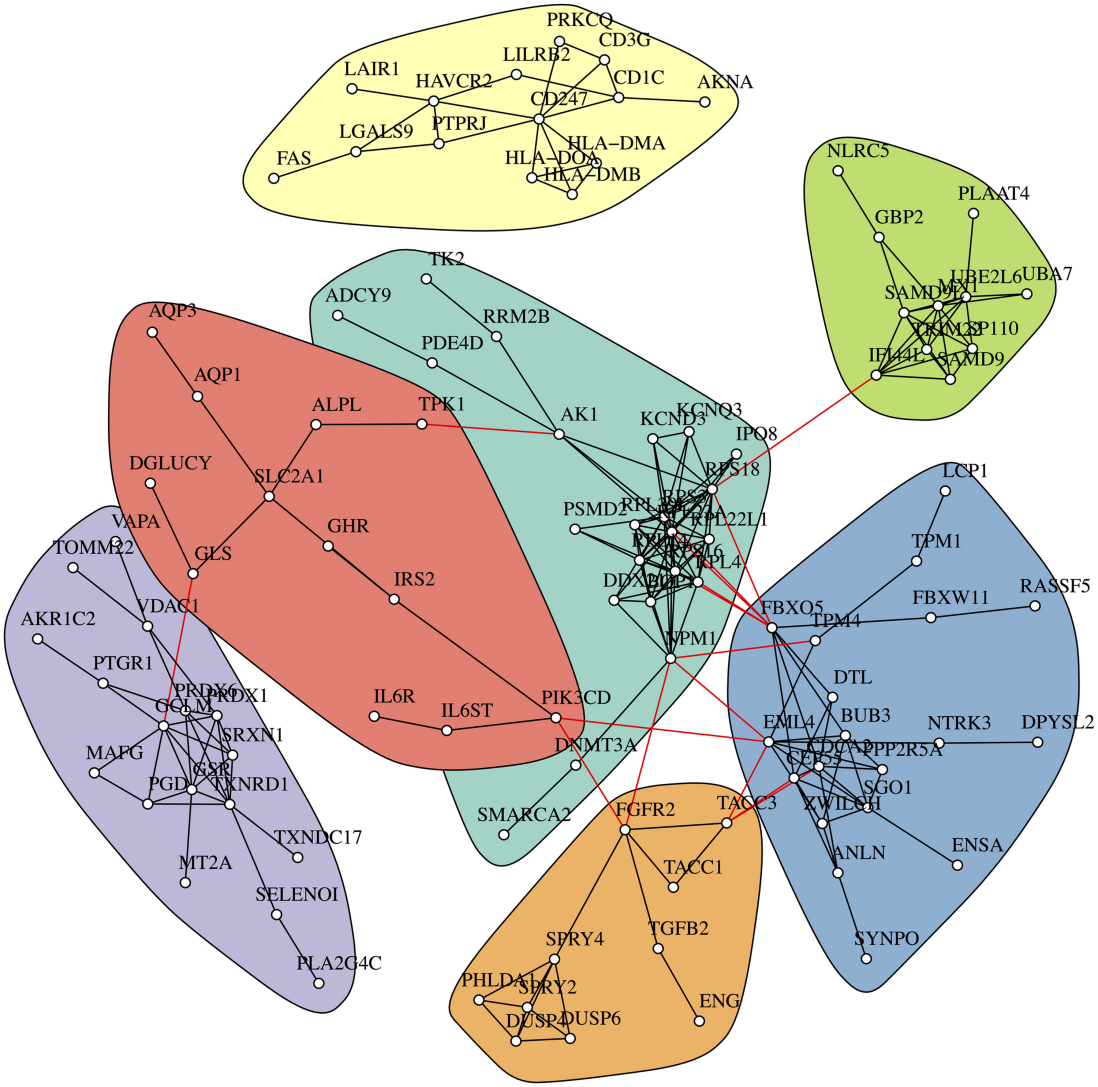
Clustering analysis of the methyl-gene dataset using the STRING database. Using the results provided by the hybrid approach, we performed a clustering analysis on the confidence scores provided by STRING. Only clusters with more than five proteins are illustrated, and the seven clusters have the same color notation as their corresponding pathways in Supplementary Fig. S13.

We applied the FDR adjustment on the nominal *P*-values provided by the proposed approaches with a cutoff of 0.1; the decorrelation and the multivariate approaches reported 739 and 602 genes, respectively. The union of the two approaches included 1212 genes; the small number of overlaps supported that the two approaches pursue different biological patterns. The hybrid approach identified 590 genes, from which 386 overlap the genes suggested by the decorrelation approach and 332 overlap the genes suggested by the multivariate approach. Then, we applied pathway analyses on the significant genes in order to confirm that the underlying pathways have a consistent biological interpretation with our prior knowledge of lung adenocarcinoma. We used the STRING (Szklarczyk *et al.* 2019; v12, beta) to analyze the results provided by the hybrid approach and identified seven main clusters in Fig. 6. Similarly, nine clusters were identified in Supplementary Fig. S12 using the union set of decorrelation and multivariate approaches. Supplementary Figures S12 and Table S3 provided a complicated diagram for genes modified by smoking-induced methylation, as we illustrated in Supplementary Fig. S13 with reference to Supplementary Fig. S14. The black network in Supplementary Fig. S13 indicates the mechanism toward apoptosis, which takes place mainly within immune-related cells, and smoking suppresses this mechanism (Wickenden *et al.* 2003). Another mechanism illustrated tumorigenesis through the red network, which takes place mainly within the epithelial cells in the airway, and smoking induces tumorigenesis through inflammation (O’Callaghan *et al.* 2010). Interestingly, comparing the subgraphs in Supplementary Fig. S13, the hybrid results seem inclined to the mechanism of suppressing apoptosis. The regulation of the apoptosis and tumorigenesis mechanisms demonstrates the conflict between tumor cells and T cells, known as the PD-1/PD-L1 pathway, and PD-L1 is often highly expressed in non–small cell lung cancer cells (Lingling *et al.* 2020). Furthermore, the methylation of promoters in the PD-1/PD-L1 pathways has been studied to realize the responses of anti-PD-1 immunotherapy in non–small cell lung cancer (Zhang *et al.* 2017, Cho *et al.* 2020). The conclusion we obtained from our analysis is not only a smoking-related gene set but mechanisms of how smoking-induced epigenetic changes were involved in the development of non–small cell lung cancer.

## 4 Conclusion

In this article, we developed the decorrelation and multivariate approaches for the purpose of using the composite test on the multimediator models. We performed extensive simulations to demonstrate the advantage of each approach: the decorrelation approach performs better when signals are consistent, and the multivariate approach provides better power when signals are diverse; compared with the joint significance and the normality-based tests, the composite test has the best power on mediation assessment in genome-wide analyses. To release the burden of multiple comparisons, we further provided a hybrid approach that captures the edges of both approaches. The utility of the proposed mediation analyses was demonstrated through their application in the analysis of data from the TCGA-LUAD and CPTAC-LUAD. Using the genes identified from the proposed statistics, we further analyzed the corresponding pathways, and our results exhibited good agreement with those of previous studies.

## Supplementary Material

btad544_Supplementary_Data

## Data Availability

The R package MACtest is available at https://github.com/roqe/MACtest.
