# Supplementary material for: Genome-wide multimediator analyses using the generalized Berk–Jones statistics with the composite test
Source: Bioinformatics. 2023 Sep 4;39(9):btad544. doi: 10.1093/bioinformatics/btad544 (PMC10500087; doi:10.1093/bioinformatics/btad544)
Supplement: btad544_Supplementary_Data [file btad544_supplementary_data.pdf]

Supplementary Materials

Genome-wide multi-mediator analyses using the generalized Berk–Jones statistics with the composite test

En-Yu Lai

Introduction

Composite null hypothesis. Studies [1, 2] have described two important issues in this scenario. First, the null hypothesis for each mechanism, $H_0 : \alpha_S = 0 \cup \beta_M = 0$, is a *composite null* of three simple nulls that are mutually exclusive, $H_0 = H_{0\emptyset} \cup H_{0\alpha} \cup H_{0\beta}$:

$$\begin{aligned} &H_{0\emptyset} : \alpha_S = 0, \beta_M = 0, \\ &H_{0\alpha} : \alpha_S \neq 0, \beta_M = 0, \\ &H_{0\beta} : \alpha_S = 0, \beta_M \neq 0. \end{aligned} \tag{1}$$

If we can not determine the proportions of each simple null, then deriving the null distribution of any test statistics is challenging. Second, the re-expression of the null hypothesis, $H_0 : \alpha_S\beta_M = 0$, reveals that the test statistics consist of the product of the coefficient estimators $\hat{\alpha}_S\hat{\beta}_M$. If the sample size is sufficient, then the estimators of the regression coefficients, $\hat{\alpha}_S$ and $\hat{\beta}_M$, behave as two random variables following a normal distribution, and their product $\hat{\alpha}_S\hat{\beta}_M$ follows a normal product distribution. Because the composite null consists of three simple nulls, the underlying null distribution is also a mixture of three types of normal product distribution, as illustrated in Figure S1(a). Notably, that the density function under $H_{0\emptyset}$ goes to infinity at zero. In other words, a large portion of the samples are centered at zero if $H_{0\emptyset}$ is the majority of H_0, and a conservative conclusion is reached when a normal approximation is used to obtain p-values. As shown in Figure S1(b), both normal approximation (Sobel) and joint significance tests are conservative, only the composite test provides uniform p-values for H_0.

Causal assumptions. We define $Y(s, \mathbf{m})$ as the *counterfactual outcome* of Y, and the random variable $Y(s, \mathbf{m})$ fully describes the behavior of Y in a possible world in which the exposure S has been set to s and the mediators M have been set to $\mathbf{m}$. Similarly, $\mathbf{M}(s)$ is defined as the counterfactual mediators in a possible world in which S has been set to s. We list the assumptions of identifiability for mediation effects as follows:

(A1) $Y(s, \mathbf{m}) \perp S \,|\, \mathbf{X}$: no unmeasured confounding between the exposure and the outcome.

(A2) $Y(s, \mathbf{m}) \perp \mathbf{M} \,|\, S, \mathbf{X}$: no unmeasured confounding between the mediators and the outcome.

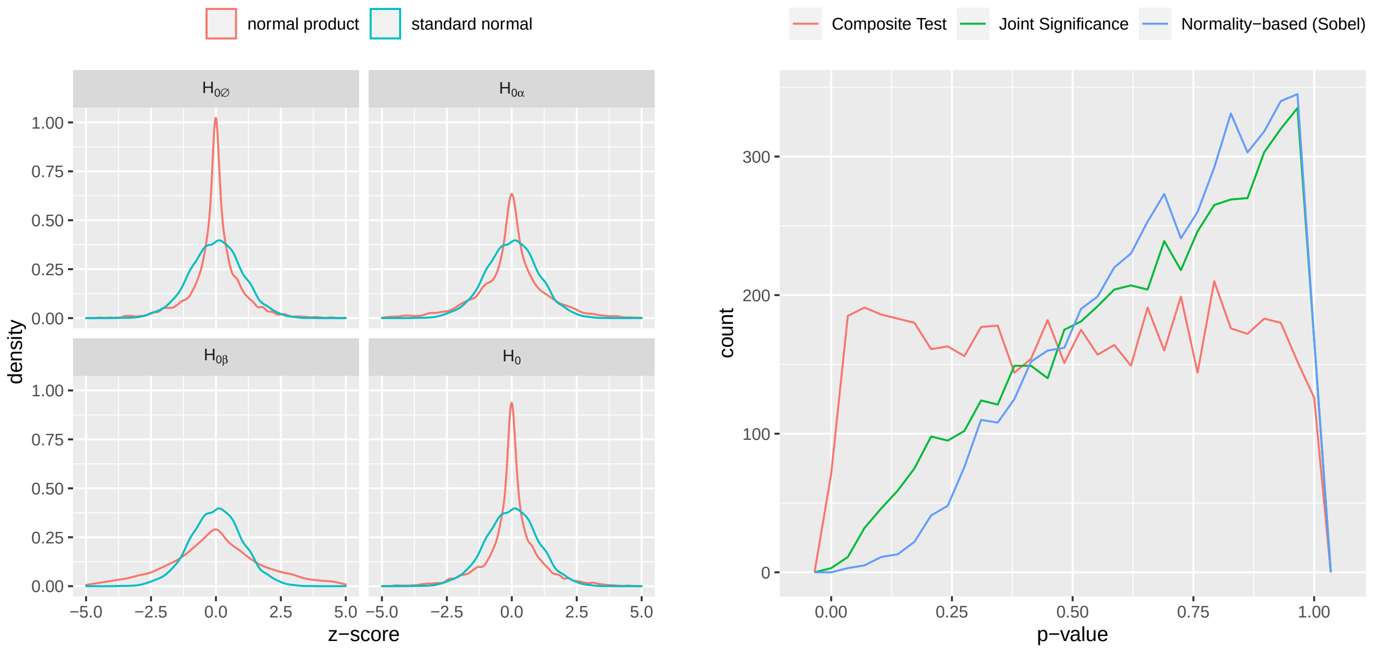

(a) Composition of the composite null distribution (b) Histogram of p-values under the composite null H_0

Figure S1: **Composite null.** (a) We demonstrate the composite null distribution using by 5000 samples of coefficient estimators, where $\alpha_S = \beta_M = 0$ under $H_{0\emptyset}$, $\alpha_S = 1$ and $\beta_M = 0$ under $H_{0\alpha}$, and $\alpha_S = 0$ and $\beta_M = -2$ under $H_{0\beta}$. We then also show a possible composite null H_0, which includes 4000 samples from $H_{0\emptyset}$, 950 samples from $H_{0\alpha}$, and 50 samples from $H_{0\beta}$. The standard normal distributions are also illustrated as a reference. Intuitively, $|z| > 1.96$ covers 5% of the area under a standard normal distribution. If $H_{0\emptyset}$ dominates H_0 and we use a standard normal distribution as the null distribution instead of the normal production distribution, then $|z| > 1.96$ only covers, for example, 3% of the area; therefore, the overall number of significant samples decreases because of poor approximation. (b) Using the simulated data of H_0, we plot the histogram of p-values using three tests for mediation assessment. Only the composite test results p-values of uniform distribution.

(A3) $\mathbf{M}(s) \perp S \,\big|\, \mathbf{X}$: no unmeasured confounding between the exposure and the mediators.

(A4) $\mathbf{M}(s*) \perp Y(s, \mathbf{m}) \,\big|\, \mathbf{X}$: no confounder between the mediator and the outcome that is affected by the exposure.

Assumptions A1, A2, and A3 suggest the presence of no unmeasured confounding other than the confounding contributed by the random variables in the regression models: the confounders between the exposure and the counterfactual mediator should be adjusted in Equation (1); the confounders between the exposure and the outcome, or between the mediator/exposure and the outcome, should be adjusted in Equation (2). The assumption A4 eliminates confounders with behavior similar to that of U in Figure S2 because their effects would alter the mediation effect of interest.

Disjoint effect and perfect cancellation. Extending the single-mediator model, we can formulate the null hypothesis for testing $\mathbb{G}^i$ as follows:

$$H_0 : \alpha_S^i = 0 \cup \beta_M^i = 0.$$

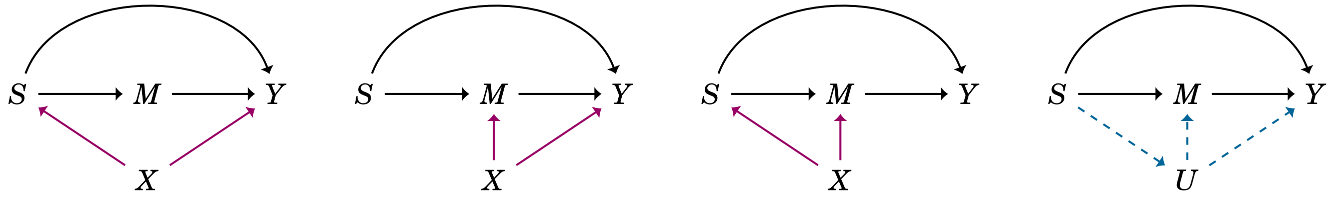

(A1) $Y(s,\mathbf{m}) \perp S \,|\, \mathbf{X}$ **(A2)** $Y(s,\mathbf{m}) \perp \mathbf{M} \,|\, S,\mathbf{X}$ **(A3)** $\mathbf{M}(s) \perp S \,|\, \mathbf{X}$ **(A4)** $\mathbf{M}(s*) \perp Y(s,\mathbf{m}) \,|\, \mathbf{X}$

Figure S2: **Causal assumptions.** Assumptions A1, A2, and A3 suggest the presence of no unmeasured confounding other than the confounding contributed by the random variables included in Equations (1) and (2). The assumption A4 suggests the absence of exposure-induced confounding. The confounding illustrated with solid edges can be adjusted in the model, but that illustrated with dashed edges should be avoided.

This null hypothesis can distinguish mediation effects in Figure S3(a) from the null in Figure S3(b). However, a special case exists in which the effect from the exposure to the mediator is not directly linked to the effect from the mediator to the outcome; this is known as the *disjoint effect*, as depicted in Figure S4. A case involving the disjoint effect is difficult to interpret because the causal relation between the mediators remains unverified, and the null hypothesis may be rejected in this scenario [3] because it is compatible with an authentic mediation effect, as indicated in Figure S4(b). Figure S4 illustrates the possible causal interpretation of correlated mediators. Notably, if the mediators are independent, then the disjoint effect case should be regarded as null (no effect). Denote the estimator of covariance, $\hat{S}^{ij} = \widehat{\text{Cov}}(M^i, M^j)$, is of zero mean and ξ variance. We assume the mediators within a predefined mechanism are all correlated in practice since the continuous random variable $P(\hat{S}^{ij} = 0) = 0$ for all $\xi > 0$. Therefore, no such mechanism with independent mediators and a disjoint effect is involved. This assumption is also biologically acceptable since the bio-molecules within a mechanism (e.g. co-regulation towards the same gene) usually work together. Another special case is the *perfect cancellation* scenario depicted in Figure S3(d), also known as the *causal faithfulness* assumption. Because the test statistic for $\mathbb{G}^i$ takes into consideration the effects from all the mediators within the mechanism, a path for a positive effect may exist with an effect magnitude identical to that of a path for a negative effect, which results in no marginal mediation effect. We assumed that no perfect cancellation exists because the focus of this study was the evaluation of any mediation effect at an element-wise level. Also, this assumption is acceptable in practice since the exact cancellation only happens with zero probability [4]. We apply this assumption only to the decorrelation approach, not to the multivariate approach.

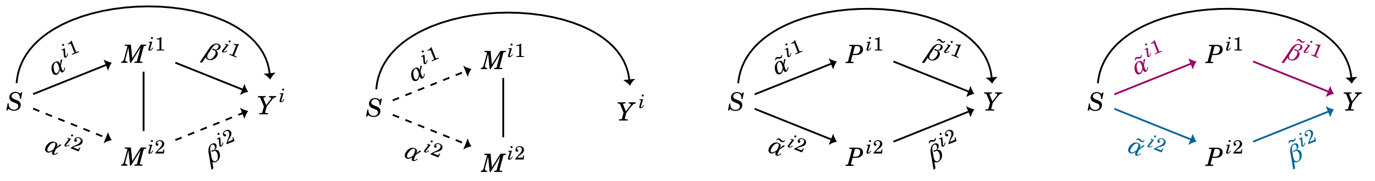

(a) Mediation effect (b) No mediation effect (c) Decorrelated mediators (d) Perfect cancellation

Figure S3: **Two-mediator model.** If no edge exists between two variables, then no association exists between them. A solid edge indicates an association and a dashed edge indicates a causal effect that may or may not exist. The effects of the purple path and the emerald path are of identical strength but are in different directions; thus, the total mediation effect is perfectly canceled.

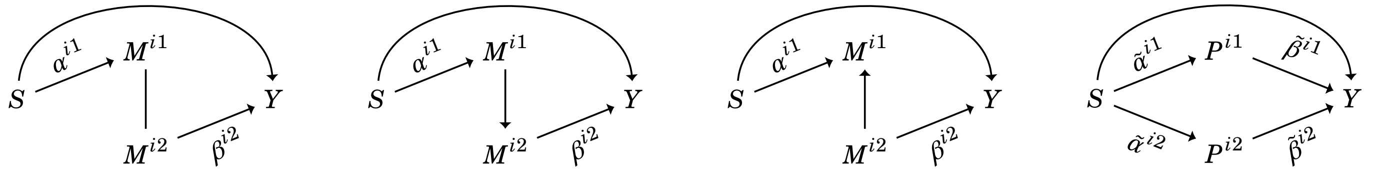

(a) Correlated mediators (b) Authentic effect (c) Spurious effect (d) Decorrelated mediators

Figure S4: **Disjoint effect.** Correlations between mediators may originate from (a) common causes, (b) causation from M^1 to M^2, or (c) causation from M^2 to M^1. However, only scenario (b) can be recognized as an authentic mediation effect. Correlated mediators M are transformed into uncorrelated mediators P when the test statistics require independence between coefficients. The mediators can be decorrelated as in (d) to prevent the causal interpretation of disjoint effects. The tilde coefficients then serve as the linear combination of the original coefficients.

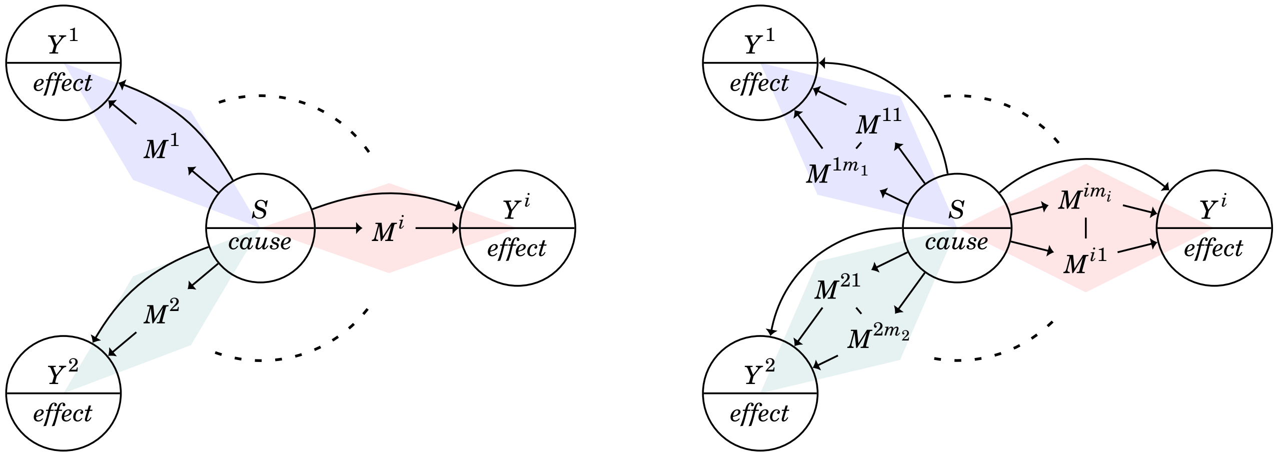

(a) Experiment involving single-mediator models (b) Experiment involving multiple-mediator models

Figure S5: **Independence at mechanism level.** Under the null hypothesis, the composite test proposed by Huang provides p-values of a uniform distribution for each mechanism in (a). The aim of the present study was to generalize this idea to multiple-mediator models in (b) to enable the evaluation of complicated mechanisms in practice. Notably, the composite test proposed by Huang requires the independence at mechanism level, or to be more precise, independence between all coefficient estimators in (a).

Method

Decorrelation of mediators. We use $\mathbf{P}^i = (P^{ij})$ to denote the vector of decorrelated mediators, $\mathbf{P}^i = \mathbf{u}^i\mathbf{M}^i$, here we use the eigen decomposition of $\hat{\epsilon}_m$ to obtain $\mathbf{u}$. The tilde coefficients are the linear combination of the original coefficients. For example, a two-mediator model allows for the construction of a 2×2 orthonormal matrix, $\mathbf{u} = \left[\begin{smallmatrix} p & q \\ r & s \end{smallmatrix}\right]$, that satisfies $\mathbf{u}\Sigma\mathbf{u}^\top = \text{diag}\{\sigma_1^2, \sigma_2^2\}$, $\epsilon_P \sim N(\mathbf{0}, \mathbf{u}\Sigma\mathbf{u}^\top)$, $\tilde{\alpha}_S = \mathbf{u}\boldsymbol{\alpha}_S = \begin{bmatrix} p\alpha_S^1 + q\alpha_S^2 \\ r\alpha_S^1 + s\alpha_S^2 \end{bmatrix}$, $\tilde{\alpha}_X = \mathbf{u}\boldsymbol{\alpha}_X$, and $\tilde{\beta}_M = \mathbf{u}\boldsymbol{\beta}_M = \begin{bmatrix} p\beta_M^1 + q\beta_M^2 \\ r\beta_M^1 + s\beta_M^2 \end{bmatrix}$. If the original effects are as detailed in Figure S3(a) (i.e., $\alpha_S^1 \neq 0, \alpha_S^2 = 0, \beta_M^1 = 0, \beta_M^2 \neq 0$), then the effects of decorrelated models will become weighted averages of original coefficients (i.e. $\tilde{\alpha}_S^1 = p\alpha_S^1, \tilde{\alpha}_S^2 = r\alpha_S^1, \tilde{\beta}_M^1 = q\beta_M^2, \tilde{\beta}_M^2 = s\beta_M^2$) and none of them are zero-valued coefficients as illustrated in Figure S3(c). The decorrelation procedure can preserve the truth value of the null hypothesis and identify disjoint effects as mediation effects.

Proof of $a^i \xrightarrow{d} N(0,1)$ under the null in the multivariate approach. For the GBJ statistics (or any multivariate statistic) in the multivariate approach, says T_α^i, we prove that the score statistic $a^i = \text{sign}(\hat{\alpha}_S^i)\,\Phi^{-1}(1 - p_\alpha^i/2)$ converges to the standard normal distribution, if the following three statements hold:

1. $\text{sign}(\hat{\alpha}_S^i)$ takes values of -1 and 1 with equal probabilities.
2. $\Phi^{-1}(1 - p_\alpha^i/2)$ converges to the absolute value of a standard normal random variable.
3. $\text{sign}(\hat{\alpha}_S^i)$ and $\Phi^{-1}(1 - p_\alpha^i/2)$ are independent asymptotically.

Part 1. The function $\text{sign}(\hat{\alpha}_S^i) = \{1 - 2\mathfrak{I}(\sum_j \hat{\alpha}_S^{ij} < 0)\}$ represents the collective direction of $\hat{\alpha}_S^{ij}$. Under the null, the maximum likelihood estimator $\sqrt{n}\hat{\alpha}_S^i$ converges to a zero-mean multivariate normal distribution with a bounded covariance; consequently, $\sqrt{n}\sum_j \hat{\alpha}_S^{ij} = \sqrt{n}\mathbf{1}^\top\hat{\alpha}_S^i$ converges to a zero-mean normal distribution with a finite variance, where $\mathbf{1}$ is a vector of one's. Since $\sqrt{n}\sum_j \hat{\alpha}_S^{ij}$ is centered at 0 and symmetric, it takes positive and negative values with equal probabilities. Therefore, $\{1 - 2\mathfrak{I}(\sum_j \hat{\alpha}_S^{ij} < 0)\}$ takes values of -1 and 1 with equal probabilities.

Part 2. Since p_α^i is the p-value of T_α^i, under the null, it converges to a random variable following a uniform distribution ranging from 0 to 1. It follows that $\Phi^{-1}(1 - p_\alpha^i/2)$ converges to the absolute value of a standard normal random variable U with a probability density function $f_U(u) = 2(2\pi)^{-1/2}e^{-u^2/2}$ and support $u \in [0,\infty)$. We denote the test statistic a^i as $V = \text{sign}(\hat{\alpha}_S^i)U$ and show that $f_{V|\text{sign}(\hat{\alpha}_S^i)}(v) = f_U(v)$ with support $v \in [0,\infty)$ if $\text{sign}(\hat{\alpha}_S^i) = 1$ and $v \in (-\infty, 0]$ if $\text{sign}(\hat{\alpha}_S^i) = -1$. The moment-generating

function of V is

$$\begin{aligned}E[e^{tv}] &= \int_{-\infty}^{\infty} e^{tv} f_V(v)dv \\ &= \int_{-\infty}^{\infty} e^{tv} f_{V|\text{sign}(\hat{\alpha}_S^i)}(v)P(\text{sign}(\hat{\alpha}_S^i)=1)dv + \int_{-\infty}^{\infty} e^{tv} f_{V|\text{sign}(\hat{\alpha}_S^i)}(v)P(\text{sign}(\hat{\alpha}_S^i)=-1)dv \\ &= \int_{0}^{\infty} e^{tv} f_U(v)P(\text{sign}(\hat{\alpha}_S^i)=1)dv + \int_{-\infty}^{0} e^{tv} f_U(v)P(\text{sign}(\hat{\alpha}_S^i)=-1)dv \\ &= \frac{1}{2}\times 2\int_{0}^{\infty} e^{tv}\frac{1}{\sqrt{2\pi}}e^{-v^2/2}dv + \frac{1}{2}\times 2\int_{-\infty}^{0} e^{tv}\frac{1}{\sqrt{2\pi}}e^{-v^2/2}dv \quad \text{[Part 1.]} \\ &= \int_{-\infty}^{\infty} e^{tv}\frac{1}{\sqrt{2\pi}}e^{-v^2/2}dv\end{aligned}$$

Part 3. Huang [5] has demonstrated that the probability of the score of α_S^i converges to the probability of the estimator $\hat{\alpha}_S^i$, i.e. $P(n^{-1}Q_\alpha < q) \to P(n||\hat{\alpha}_S^i||^2 < q)$. Also, we know that consider the event of $\text{sign}(\hat{\alpha}_S^i)$ is equivalent to the event of $\sqrt{n}\mathbf{1}^\top\hat{\alpha}_S^i$ in Part 1, so next we show $\sqrt{n}\mathbf{1}^\top\hat{\alpha}_S^i < 0$ and $n||\hat{\alpha}_S^i||^2$ are asymptotically independent. Since $\sqrt{n}\hat{\alpha}_S^i$ converges to a random variable ζ_α following a multivariate normal distribution, $n||\hat{\alpha}_S^i||^2 < q$ indicates the integral of ζ_α's pdf within the multivariate hemispherical shape $n||\hat{\alpha}_S^i||^2 = q$. Also notice that ζ_α's pdf is a symmetric function centered at the origin, so $\sqrt{n}\mathbf{1}^\top\hat{\alpha}_S^i = 0$ becomes the hyperplane that splits ζ_α's pdf into two symmetric spaces through the origin (a demonstrative figure can be found at [5], Figure S6). Therefore we know

$$P\big(n||\hat{\alpha}_S^i||^2 < q \mid \sqrt{n}\mathbf{1}^\top\hat{\alpha}_S^i < 0\big) = P\big(n||\hat{\alpha}_S^i||^2 < q \mid \sqrt{n}\mathbf{1}^\top\hat{\alpha}_S^i \geq 0\big). \tag{2}$$

We can further show that

$$\begin{aligned}P\big(n||\hat{\alpha}_S^i||^2 < q\big) &= P\big(n||\hat{\alpha}_S^i||^2 < q \mid \sqrt{n}\mathbf{1}^\top\hat{\alpha}_S^i < 0\big)P(\sqrt{n}\mathbf{1}^\top\hat{\alpha}_S^i < 0) \\ &\quad + P\big(n||\hat{\alpha}_S^i||^2 < q \mid \sqrt{n}\mathbf{1}^\top\hat{\alpha}_S^i \geq 0\big)P(\sqrt{n}\mathbf{1}^\top\hat{\alpha}_S^i \geq 0) \\ &= P\big(n||\hat{\alpha}_S^i||^2 < q \mid \sqrt{n}\mathbf{1}^\top\hat{\alpha}_S^i < 0\big)\frac{1}{2} + P\big(n||\hat{\alpha}_S^i||^2 < q \mid \sqrt{n}\mathbf{1}^\top\hat{\alpha}_S^i \geq 0\big)\frac{1}{2} \quad \text{[Part 1.]} \\ &= P\big(n||\hat{\alpha}_S^i||^2 < q \mid \sqrt{n}\mathbf{1}^\top\hat{\alpha}_S^i < 0\big)\frac{1}{2} + P\big(n||\hat{\alpha}_S^i||^2 < q \mid \sqrt{n}\mathbf{1}^\top\hat{\alpha}_S^i < 0\big)\frac{1}{2} \quad \text{[Equation 2]} \\ &= P\big(n||\hat{\alpha}_S^i||^2 < q \mid \sqrt{n}\mathbf{1}^\top\hat{\alpha}_S^i < 0\big)\end{aligned}$$

Therefore, we establish the independence of $n||\hat{\alpha}_S^i||^2$ and the event $\sqrt{n}\mathbf{1}^\top\hat{\alpha}_S^i < 0$. Since $\text{sign}(\hat{\alpha}_S^i)$ and $\Phi^{-1}(1-p_\alpha^i/2)$ are functions of $\sqrt{n}\mathbf{1}^\top\hat{\alpha}_S^i < 0$ and $n||\hat{\alpha}_S^i||^2$, respectively, they are asymptotically independent.

A similar result can be obtained for $b^i = \text{sign}(\hat{\beta}_M^i)\,\Phi^{-1}(1-p_\beta^i/2)$.

Results

Simulation: Type I errors

We demonstrate that our p-value calculations are sufficiently accurate for controlling the type I error rate. Under the null scenario, the p-values should follow a uniform distribution. We simulated three

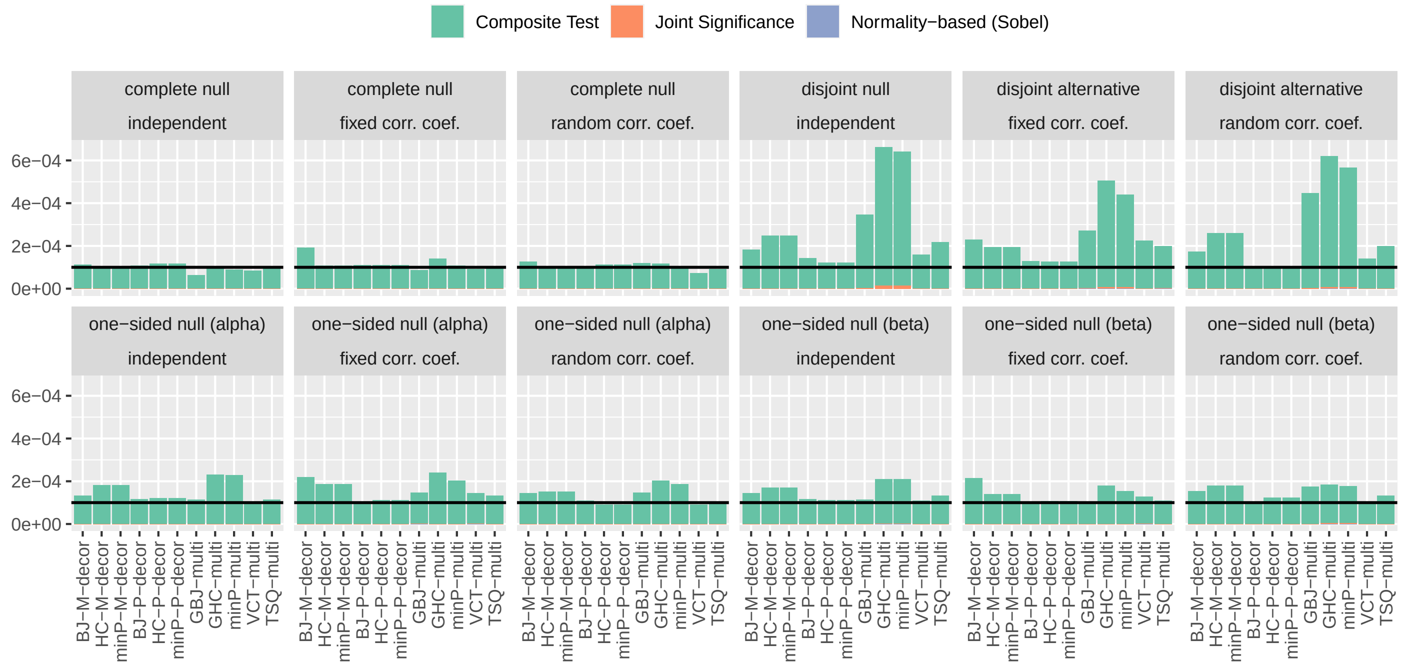

Figure S6: **Type I error using the composite test, joint significance test, and normality-based test.** With a sufficient sample size ($n = 1000$) for estimating the coefficients, we could successfully control the type I error by using a multivariate approach or decorrelation approach with a decorrelation process (i.e., approaches using P as mediators, as described in Equation (7)). The adoption of a decorrelation approach without a decorrelation process (i.e., approaches in which M is used as a mediator, as described in Equation (1)) may lead to an inflated type I error. Disjoint cases are recognized as alternative scenarios using multivariate approaches. We use the significance level $\alpha = 0.0001$ since the analysis is supposed to be applied on gene level.

types of null scenarios, as described in Table S1(a). The first was complete null scenarios, which only consisted of $H_{0\emptyset}$, and resulted in the poor approximation of the normal product distribution. The second was one-sided null scenarios, in which some mechanisms (10% of 10000 mechanisms) have effects only in the direction from the exposure to the mediators or from the mediators to the outcome. The third was disjoint null scenarios, which refer to scenarios that were omitted from our model settings, namely those with disjoint effects without a correlation between mediators within one mechanism. As mentioned, we omitted these scenarios because they would be recognized as alternative cases under the application of a multivariate approach. The results using composite test were summarized in Table S2, and the scenario of nominal $\alpha = 0.0001$ was illustrated in Figure S6 to compare the three tests for mediation assessment.

Simulation: Power

Different mediator dependency settings and signal patterns. Figure S7 indicates that skipping decorrelation outperformed the multivariate approaches for the alternative cases with independent mediators (first row), and they could identify the disjoint cases from the connected cases (the first row vs.

30 mediators in each $\mathbb{G}^i$ (300 experiments performed)	Complete null (only $H_{0\emptyset}$)	One-sided null ($H_{0\emptyset} \cup H_{0\alpha}$ or $H_{0\emptyset} \cup H_{0\beta}$)	Disjoint null ($H_{0\emptyset} \cup H_{0\alpha} \cup H_{0\beta}$)
Sample size (n)	100 or 1000	1000	1000
Dependency among mediators (ρ)	independent, fixed, or random	fixed	independent
Number of signals in one side (h)	0	1 (10%)	1 (10%)
Mean of signals (μ)	-	0	0
Standard deviation of signals (v)	-	0.05	0.05

(a) Type I error

m mediators in each $\mathbb{G}^i$ (e experiments performed)	Alternative ($m = 30, e = 30$)	Disjoint alternative ($m = 30, e = 30$)	Alternative ($m = 100, e = 10$)
Sample size (n)	1000	1000	3000
Dependency among mediators (ρ)	independent, fixed, or random	fixed	independent or fixed
Number of signals in one side (h)	2 to 10 (10%, 50%)	2 to 10 (10%)	2 to 10 (10%, 100%)
Mean of signals (μ)	0.05	0.05	0 or 0.05
Standard deviation of signals (v)	0.1	0.1	0 or 0.1

(b) Power

Table S1: **Simulation settings.** General settings for one experiment consist of 10000 mechanisms, with each containing 30 mediators. Three dependency settings were applied, *independent*, *fixed* (a block of 10 mediators with the correlation coefficients fixed to 0.3), and *random* (three blocks of mediators are correlated; the block sizes are two, three, five, with random correlation coefficients sampled from a uniform distribution of different maximum bound, 0.8, 0.5, 0.3, respectively; then the simulated correlation matrix will be adjusted to be positive definite). (a) We modified the general settings to assess the impact of the sample size, application of a decorrelation process, and null type. (b) We investigated the effects of sparsity level, signal strength, signal pattern, and the number of mechanisms on the power of the proposed statistics.

the fourth row). This result is attributable to the fact that the decorrelation approach can preserve the relationships between coefficients for each mediator, whereas the multivariate approaches regarded the mediators as a group. However, the decorrelation process based on sample covariance disturbs the relationships; consequently, identifying disjoint cases is equally as tricky with decorrelation approaches involving a decorrelation process as it is with multivariate approaches (the second row vs. the fifth row). We also found that the scenario of random correlation coefficients has lower power than the fixed scenario; the result suggests that higher mediator dependency may decrease the power of the tests (the second row vs. the third row).

Different signal strengths and sparsity levels. Sun and Lin suggested that the best performance is achieved with minP and GHC in cases with extremely sparse data ($h \leq 3$; i.e., in our settings of $m = 100$), GBJ in cases with moderately sparse data ($4 \leq h \leq 10$), and VCT in cases with dense signals ($\mu = 0.05, v = 0$, 100% active mechanisms in Figure S8 is the most similar setting to that adopted by Sun and Lin). Notably, Sun and Lin only provided statistics for evaluating one mechanism and did not consider the genome-wide background; thus, their simulation produced scenarios of 100% active mechanisms. As discussed, a simulation in which 100% of mechanisms are assumed to have signals is unrealistic, and the conclusions drawn from the results obtained with such a setting may be inapplicable to real data analysis. Besides, the scenario provided by Sun and Lin is not a mediation design, their boundaries of the dominant regimes also changed; therefore, in practice, the highest-performing statistic is difficult to conclusive determine. However, following Figure S7 and Figure S8, general conclusions can be drawn:

1. The decorrelation approach performs better when signals are consistent, similar for the multivariate approach when signals are diverse (VCT and TSQ do not have corresponding decorrelation approaches, so this conclusion is based on the other three tests).

2. GHC and the global minP test achieved the best performance in the extremely sparse region, TSQ is generally the most powerful in the moderately sparse region; VCT and decorrelation approaches outperformed GBJ, GHC, and global minP tests when the signals were consistent.

3. The hybrid approach is able to capture the better results among the decorrelation and multivariate approach without raising the more multiple comparison issue.

To summarize our simulations, preferences of the test statistics are presented in Figure S9.

nominal α level	hypothesis	mediator setting	*decorrelation approach* original mediators BJ	HC	minP	decorrelated mediators BJ	HC	minP	*multivariate approach* original mediators VCT	TSQ	GBJ	GHC	minP
0.05	complete	indp.	0.0496	0.0502	0.0502	0.0494	0.0504	0.0507	0.0496	0.0498	0.0411	0.0500	0.0498
		corr./$_{\text{fixed}}$	0.0530	0.0508	0.0498	0.0495	0.0505	0.0506	0.0495	0.0499	0.0382	0.0502	0.0497
		corr./$_{n=100}$	0.0609	0.0566	0.0539	0.0506	0.0512	0.0509	0.0473	**0.0695**	0.0498	0.0508	0.0505
		corr./$_{\text{random}}$	0.0502	0.0501	0.0497	0.0492	0.0501	0.0502	0.0497	0.0496	0.0497	0.0499	0.0496
	disjoint	indp.	0.0502	0.0521	0.0523	0.0505	0.0511	0.0509	0.0503	0.0511	0.0420	0.0490	0.0490
		corr.	0.0534	0.0523	0.0515	0.0501	0.0509	0.0507	0.0501	0.0510	0.0390	0.0495	0.0490
	one-sided (alpha)	indp.	0.0498	0.0511	0.0511	0.0498	0.0510	0.0508	0.0494	0.0496	0.0409	0.0488	0.0486
		corr.	0.0531	0.0517	0.0510	0.0494	0.0504	0.0505	0.0487	0.0494	0.0383	0.0492	0.0486
	one-sided (beta)	indp.	0.0497	0.0508	0.0509	0.0494	0.0504	0.0504	0.0495	0.0500	0.0410	0.0491	0.0488
		corr.	0.0529	0.0513	0.0505	0.0496	0.0508	0.0508	0.0489	0.0500	0.0382	0.0497	0.0491
0.01	complete	indp.	0.0100	0.0101	0.0100	0.0100	0.0103	0.0103	0.0097	0.0099	0.0075	0.0102	0.0099
		corr./$_{\text{fixed}}$	0.0119	0.0101	0.0100	0.0101	0.0104	0.0104	0.0098	0.0099	0.0073	0.0108	0.0099
		corr./$_{n=100}$	**0.0162**	0.0123	0.0120	0.0109	0.0108	0.0107	0.0075	**0.0164**	0.0119	0.0126	0.0106
		corr./$_{\text{random}}$	0.0105	0.0100	0.0100	0.0100	0.0102	0.0102	0.0096	0.0098	0.0103	0.0104	0.0099
	disjoint	indp.	0.0107	0.0115	0.0115	0.0105	0.0105	0.0104	0.0104	0.0110	0.0089	0.0118	0.0116
		corr.	0.0122	0.0112	0.0111	0.0104	0.0106	0.0106	0.0108	0.0109	0.0084	0.0119	0.0109
	one-sided (alpha)	indp.	0.0103	0.0107	0.0107	0.0102	0.0104	0.0105	0.0097	0.0099	0.0078	0.0103	0.0102
		corr.	0.0121	0.0109	0.0108	0.0099	0.0103	0.0102	0.0099	0.0100	0.0077	0.0111	0.0101
	one-sided (beta)	indp.	0.0103	0.0108	0.0108	0.0101	0.0103	0.0104	0.0097	0.0103	0.0077	0.0103	0.0102
		corr.	0.0120	0.0106	0.0105	0.0100	0.0106	0.0105	0.0100	0.0102	0.0075	0.0109	0.0099

continued on next page

Table S2 – continued from previous page

nominal α level	hypothesis	mediator setting	original mediators			decorrelated mediators			original mediators				
			BJ	HC	minP	BJ	HC	minP	VCT	TSQ	GBJ	GHC	minP
0.001	complete	indp.	0.0011	0.0010	0.0010	0.0010	0.0011	0.0011	0.0009	0.0010	0.0006	0.0011	0.0010
		corr./$_{fixed}$	**0.0015**	0.0010	0.0010	0.0011	0.0011	0.0011	0.0009	0.0010	0.0007	0.0012	0.0010
		corr./$_{n=100}$	**0.0026**	**0.0015**	**0.0015**	0.0013	0.0011	0.0011	0.0005	**0.0021**	**0.0016**	**0.0017**	0.0011
		corr./$_{random}$	0.0011	0.0010	0.0010	0.0010	0.0011	0.0011	0.0008	0.0010	0.0011	0.0011	0.0010
	disjoint	indp.	0.0013	**0.0015**	**0.0015**	0.0012	0.0011	0.0011	0.0012	**0.0014**	**0.0014**	**0.0022**	**0.0021**
		corr.	**0.0016**	0.0013	0.0013	0.0011	0.0011	0.0011	**0.0014**	0.0013	0.0012	**0.0020**	**0.0018**
	one-sided (alpha)	indp.	0.0011	0.0013	0.0013	0.0011	0.0010	0.0010	0.0010	0.0010	0.0008	0.0013	0.0013
		corr.	**0.0016**	0.0013	0.0013	0.0010	0.0011	0.0011	0.0011	0.0011	0.0010	**0.0015**	0.0013
	one-sided (beta)	indp.	0.0011	0.0013	0.0013	0.0010	0.0011	0.0011	0.0010	0.0011	0.0008	0.0013	0.0013
		corr.	**0.0015**	0.0012	0.0012	0.0010	0.0011	0.0011	0.0011	0.0011	0.0008	0.0013	0.0011
1e-04	complete	indp.	1.12e-04	1.02e-04	1.02e-04	1.08e-04	1.18e-04	1.18e-04	8.40e-05	9.50e-05	6.30e-05	9.70e-05	9.00e-05
		corr./$_{fixed}$	**1.93e-04**	1.07e-04	1.07e-04	1.10e-04	1.11e-04	1.11e-04	9.10e-05	1.03e-04	8.70e-05	**1.42e-04**	1.08e-04
		corr./$_{n=100}$	**4.27e-04**	**1.83e-04**	**1.83e-04**	**1.63e-04**	1.29e-04	1.29e-04	3.10e-05	**2.61e-04**	**2.40e-04**	**2.13e-04**	1.28e-04
		corr./$_{random}$	1.26e-04	9.80e-05	9.77e-05	1.05e-04	1.13e-04	1.13e-04	7.23e-05	9.17e-05	1.20e-04	1.18e-04	1.01e-04
	disjoint	indp.	**1.83e-04**	**2.50e-04**	**2.49e-04**	**1.43e-04**	1.21e-04	1.21e-04	**1.59e-04**	**2.18e-04**	**3.47e-04**	**6.63e-04**	**6.42e-04**
		corr.	**2.29e-04**	**1.94e-04**	**1.95e-04**	1.28e-04	1.27e-04	1.26e-04	**2.26e-04**	**1.98e-04**	**2.71e-04**	**5.06e-04**	**4.41e-04**
	one-sided (alpha)	indp.	1.31e-04	**1.83e-04**	**1.83e-04**	1.17e-04	1.19e-04	1.19e-04	9.30e-05	1.13e-04	1.13e-04	**2.31e-04**	**2.29e-04**
		corr.	**2.20e-04**	**1.86e-04**	**1.86e-04**	1.00e-04	1.12e-04	1.12e-04	**1.44e-04**	1.32e-04	**1.47e-04**	**2.40e-04**	**2.03e-04**
	one-sided (beta)	indp.	**1.43e-04**	**1.69e-04**	**1.69e-04**	1.16e-04	1.11e-04	1.11e-04	1.09e-04	1.32e-04	1.14e-04	**2.10e-04**	**2.10e-04**
		corr.	**2.13e-04**	1.39e-04	1.39e-04	9.90e-05	1.06e-04	1.06e-04	1.28e-04	1.09e-04	1.05e-04	**1.79e-04**	1.54e-04

continued on next page

Table S2 – continued from previous page

nominal α level	hypothesis	mediator setting	original mediators			decorrelated mediators			original mediators				
			BJ	HC	minP	BJ	HC	minP	VCT	TSQ	GBJ	GHC	minP
1e-05	complete	indp.	**1.45e-05**	**1.45e-05**	**1.45e-05**	1.10e-05	1.40e-05	1.40e-05	8.00e-06	1.10e-05	5.50e-06	1.15e-05	9.00e-06
		corr./fixed	**2.47e-05**	1.37e-05	1.37e-05	1.27e-05	1.27e-05	1.27e-05	1.13e-05	1.10e-05	1.23e-05	**1.73e-05**	1.13e-05
		corr./n = 100	**7.80e-05**	**2.53e-05**	**2.53e-05**	**1.90e-05**	1.37e-05	1.37e-05	3.00e-07	**3.43e-05**	**3.80e-05**	**2.77e-05**	**1.67e-05**
		corr./random	1.37e-05	7.30e-06	7.30e-06	1.17e-05	1.30e-05	1.30e-05	6.30e-06	8.00e-06	1.33e-05	**1.40e-05**	1.10e-05
	disjoint	indp.	**3.25e-05**	**5.70e-05**	**5.70e-05**	**1.80e-05**	**1.65e-05**	**1.65e-05**	**3.50e-05**	**4.50e-05**	**1.13e-04**	**2.62e-04**	**2.54e-04**
		corr.	**3.93e-05**	**3.67e-05**	**3.67e-05**	**1.57e-05**	**1.73e-05**	**1.73e-05**	**4.33e-05**	**3.77e-05**	**7.50e-05**	**1.69e-04**	**1.53e-04**
	one-sided (alpha)	indp.	**2.10e-05**	**3.40e-05**	**3.40e-05**	7.50e-06	1.20e-05	1.20e-05	**1.40e-05**	**1.55e-05**	**2.20e-05**	**5.45e-05**	**5.20e-05**
		corr.	**3.77e-05**	**3.17e-05**	**3.17e-05**	9.00e-06	1.07e-05	1.07e-05	**2.33e-05**	**1.53e-05**	**2.83e-05**	**5.37e-05**	**4.50e-05**
	one-sided (beta)	indp.	**2.30e-05**	**3.50e-05**	**3.50e-05**	1.15e-05	1.25e-05	1.25e-05	**1.70e-05**	**2.20e-05**	**1.50e-05**	**4.35e-05**	**4.25e-05**
		corr.	**3.10e-05**	**1.90e-05**	**1.90e-05**	8.70e-06	8.00e-06	8.00e-06	**1.73e-05**	1.20e-05	**1.80e-05**	**2.93e-05**	**2.37e-05**

Table S2: **Type I error using the composite test.** In general we control the type I error to the significance level $\alpha = 10^{-5}$ using the multivariate approaches and decorrelation approaches with decorrelation, with exception in the following case: (1) disjoint case will be recognized as alternative scenarios (red cells) for both global and decorrelation approaches with decorrelation; (2) insufficient sample size (blue cells) also induce inflated type I error, T^2-statistic is the most vulnerable and VCT is the most robust; (3) to multivariate approaches, the one-sided null in our setting becomes a strong signal when $\alpha < 10^{-4}$, so the type I error converges to α in a slower pace than the complete null; (4) the type I error of GHC tends to inflate in the setting of correlated mediators since it presume a weaker correlation structure; (5) using the random correlation coefficient setting (green cells) generates a similar results to the fixed setting.

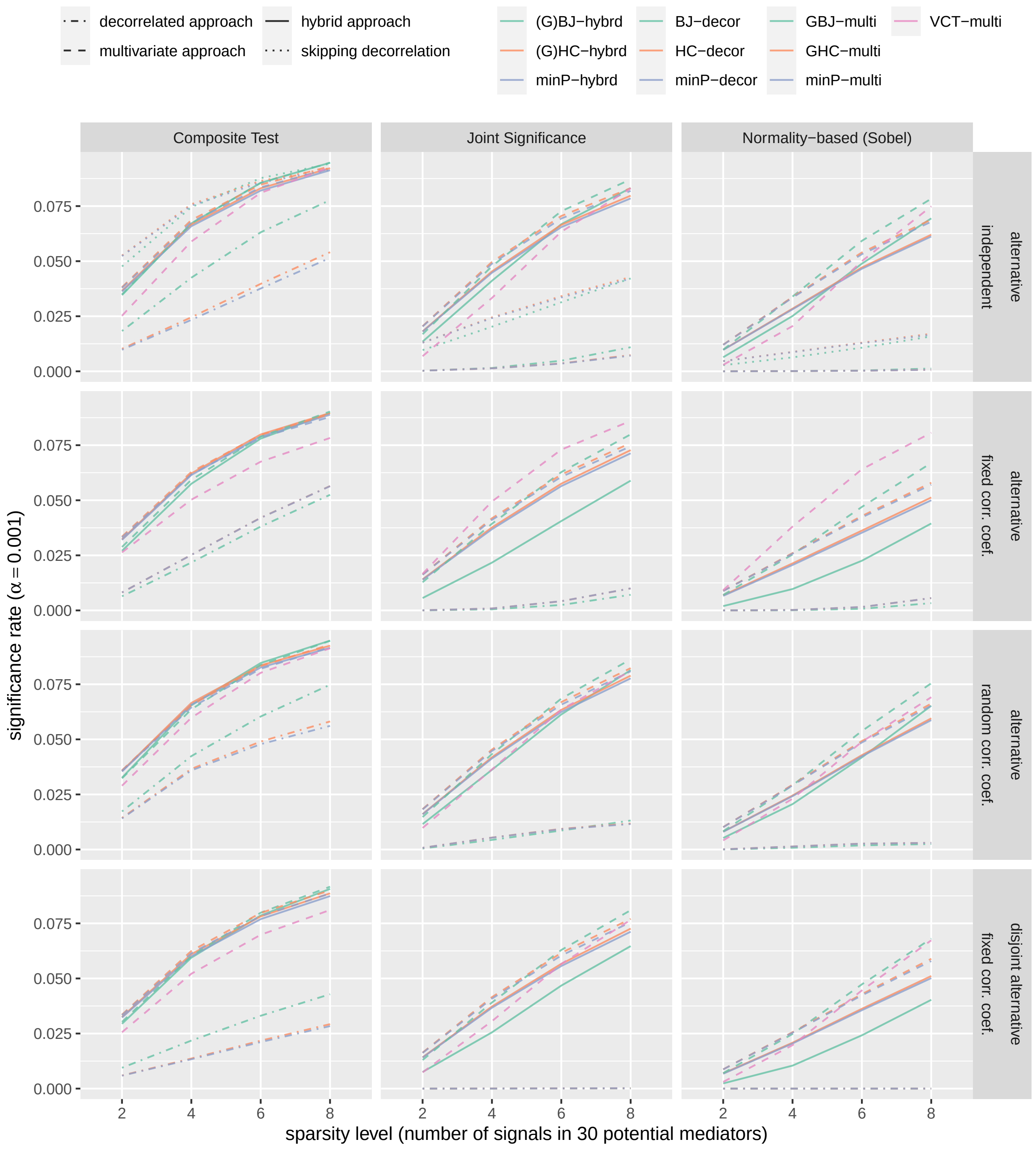

Figure S7: **Comparison of three mediation assessment approaches.** We compared eight test statistics with three mediation assessment approaches; the signal mean was fixed at 0.05, and the variance was fixed at 0.1. The significance level was 0.001 and the percentage of active mechanism is 0.1. The composite test achieved higher power when the signals were sparse, the multivariate approach is generally more powerful than the decorrelation approach, and the hybrid approach achieves comparable power toward the multivariate approach.

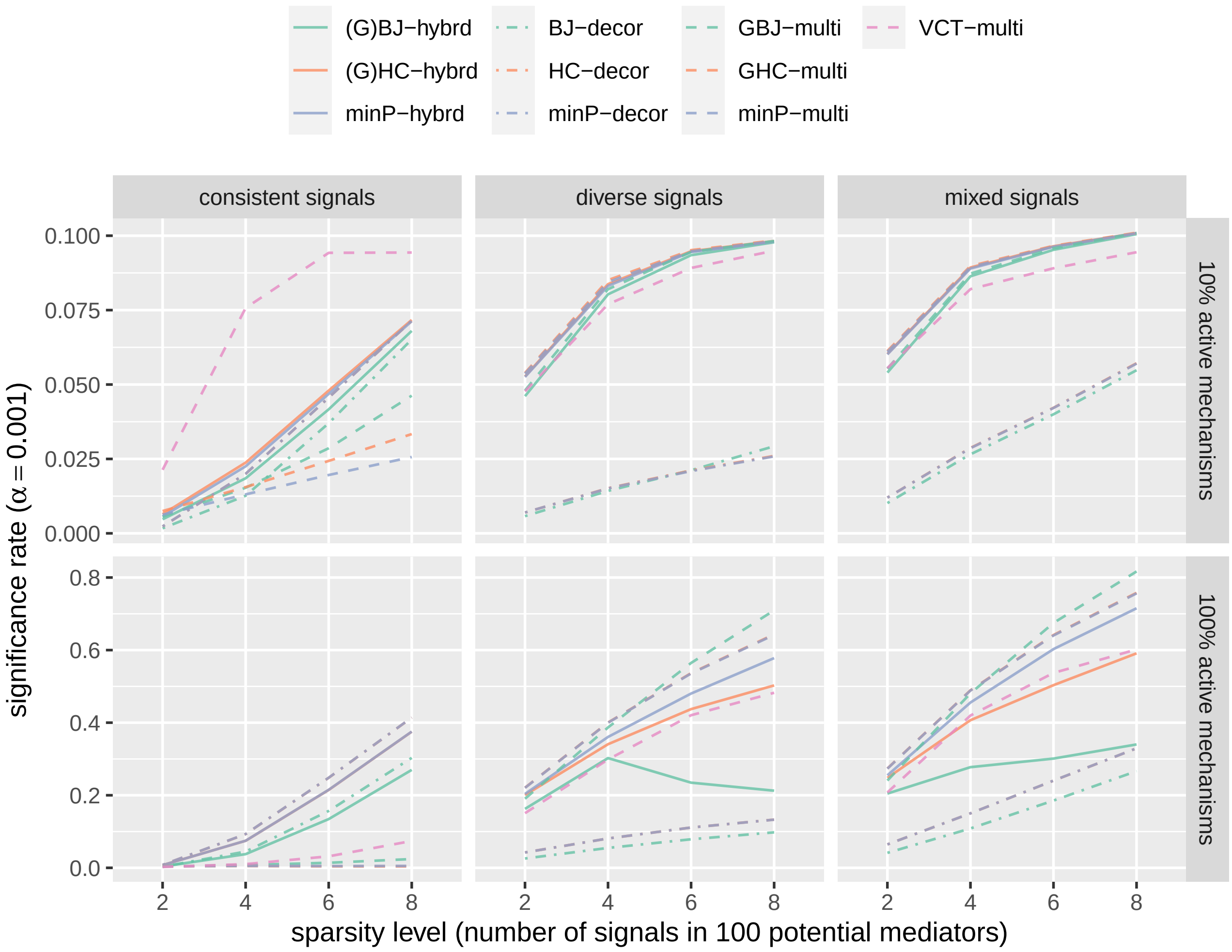

Figure S8: **Performance of the test statistics with increasing signal sparsity.** We compared eight test statistics by using the composite test with different settings for the signal patterns. The significance level was set at 0.001. The figure displays the percentage of significant mechanisms under different mediator settings and signal strengths with increasing sparsity levels.

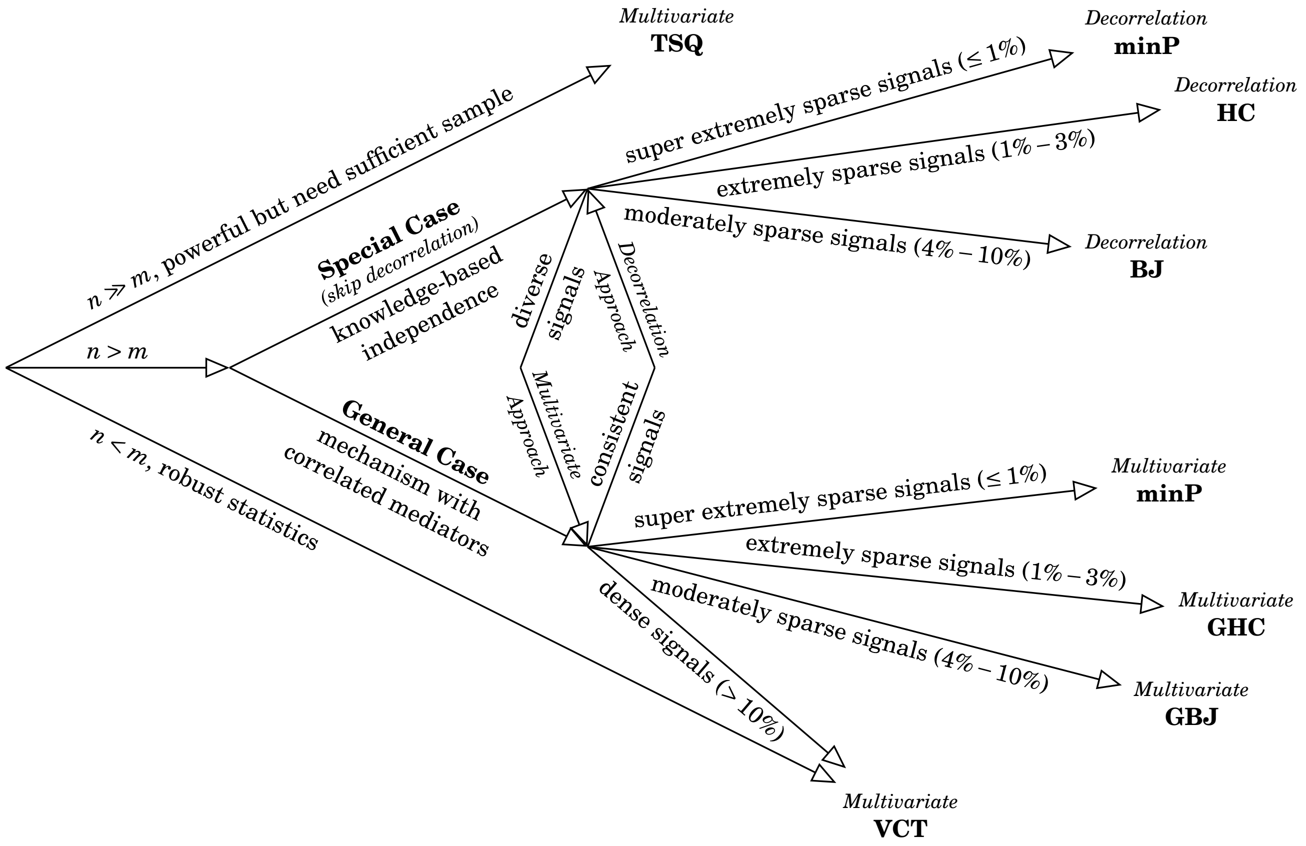

Figure S9: **Preference of the test statistics.** The preference of test statistics on the basis of signal strength and signal patterns, as well as the comparison of sample size and the number of mediators. Empirically, the sparsity can be observed through the Q–Q plots (one can even use a KS test to tell the signal strength), and the signal pattern can be observed through the histogram of ab (compare the area $ab > 0$ and $ab < 0$).

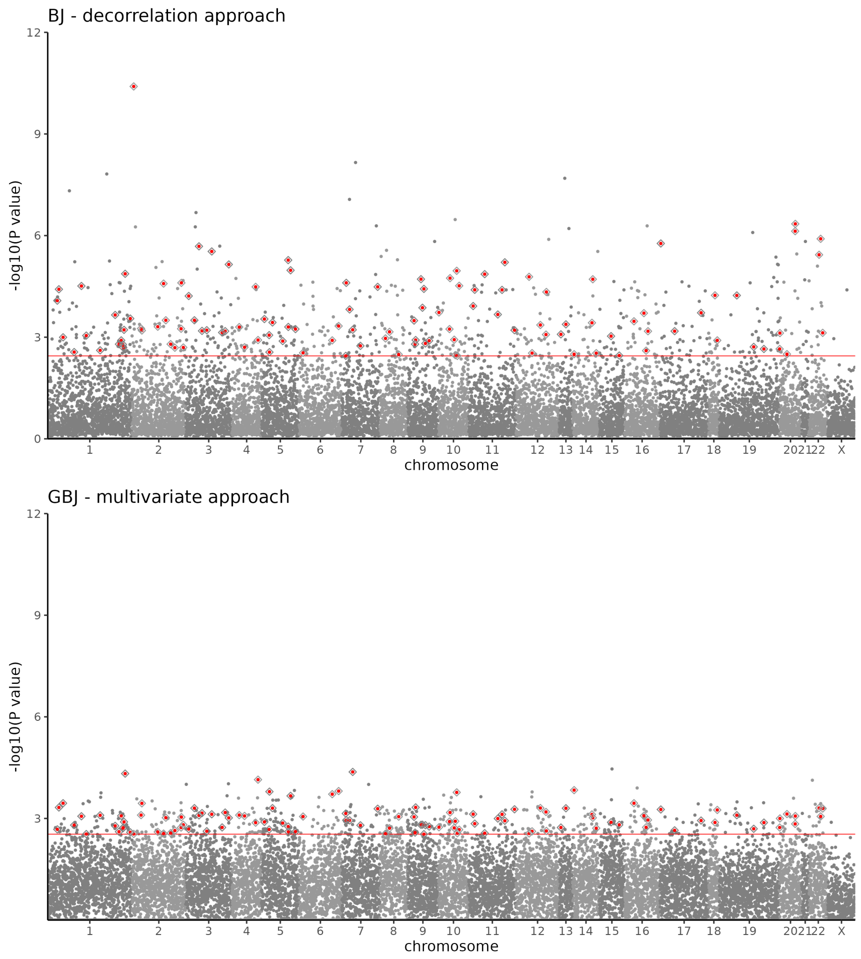

Figure S10: **Manhattan plots highlighting the significant genes in the methyl-gene dataset.** Using the composite test, the red horizontal line indicates the significance cutoff of FDR-adjusted p-value equals to 0.1; the common significant genes among the decorrelation and multivariate approaches are also highlighted.

Data application

TCGA-LUAD preprocessing The R package `TCGAbiolinks` [6] was used to retrieve the dataset from the Data Portal of Genomic Data Commons (GDC, v26.0) [7]. We filtered out the CpG loci with missing values, genes with abundant missing information (≥20%), and genes annotated with a single methylation site (i.e., $m_i = 1$) and only used the expression of the *primary tumor*. We obtained the logit and log transformation of the DNA methylation β values and gene expression values, respectively. Finally, we had 20670 genes in the *methyl-gene* dataset, and the largest mechanism consisted of 1015 potential mediators.

CPTAC-LUAD preprocessing We downloaded the normalized dataset from the supplementary material of the original paper [8]. We filtered out the miRNAs and proteins with abundant missing information (≥20%) and proteins annotated with a single miRNA, and weonly used the expression of the *tumor*. Finally, we had 6955 proteins in the *miRNA-protein* dataset and the largest mechanism consisted of 132 potential mediators.

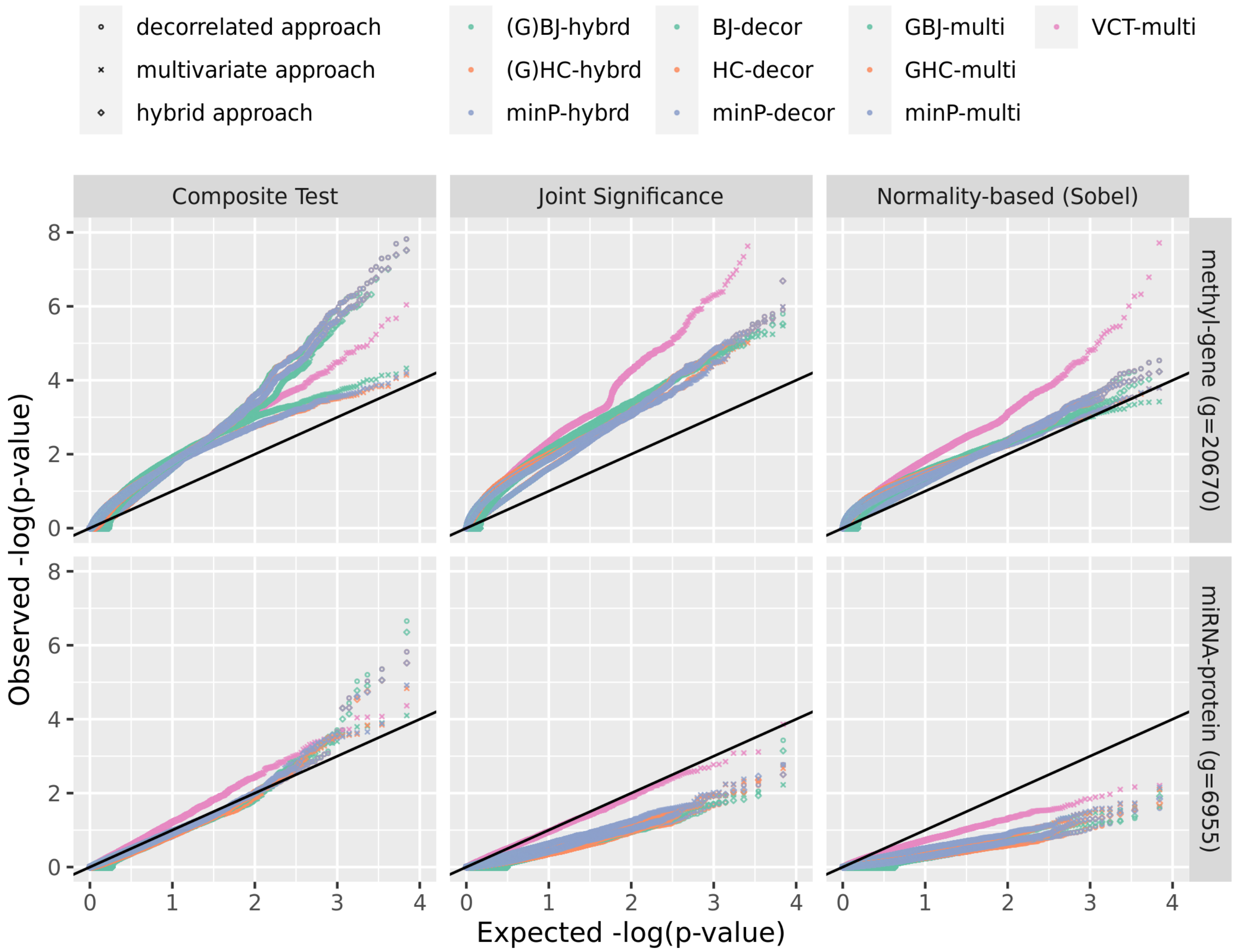

Figure S11: **Q–Q plots of all statistics.** We found that the methyl-gene dataset presented strong signals at a genome-wide scale, but the miRNA-protein dataset only reported a few proteins This plot excluded a few extreme p-values for the clarity of visualization.

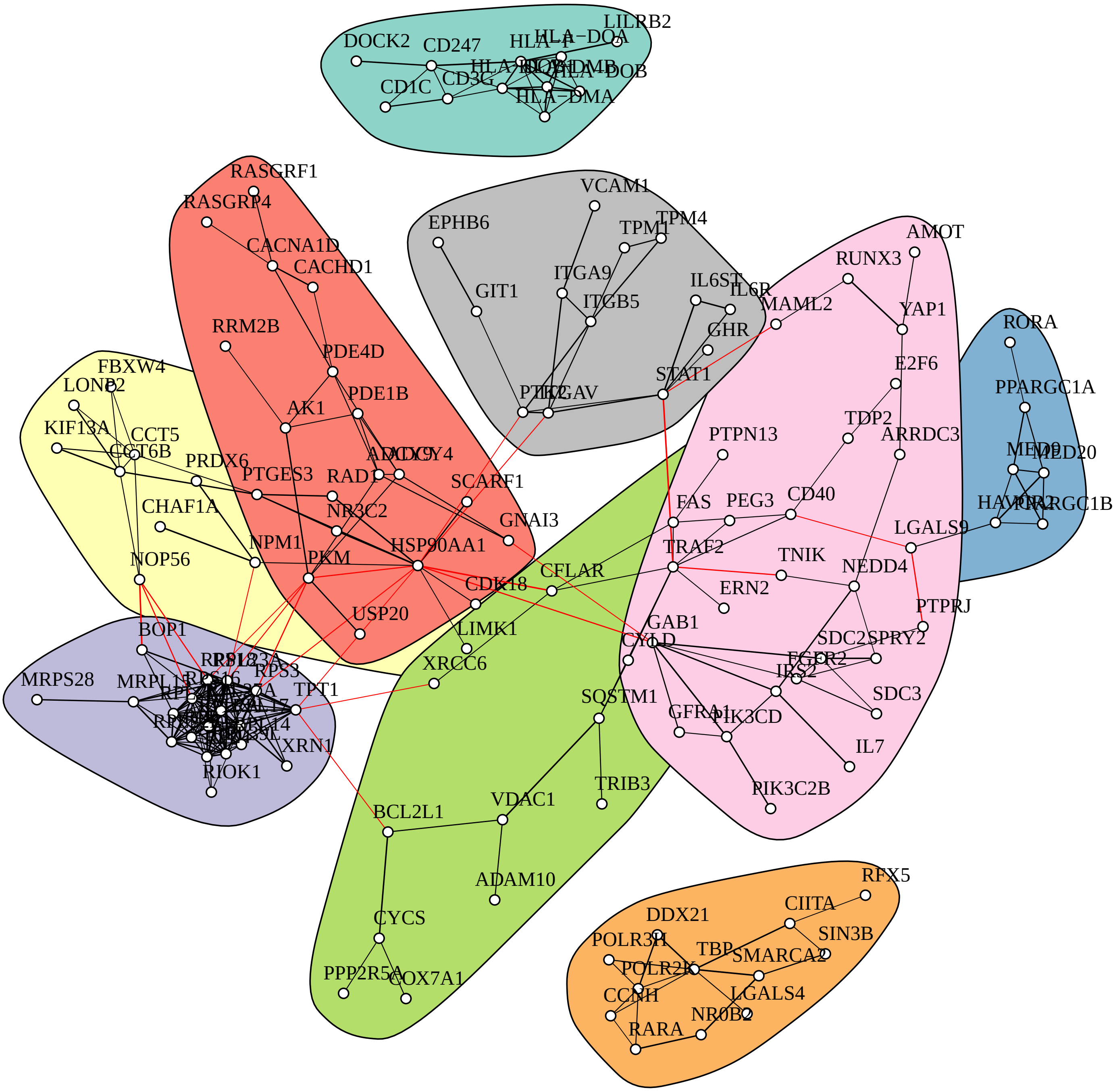

Figure S12: **Clustering analysis of the methyl-gene dataset using the STRING database.** Using the gene sets provided by the decorrelation and multivariate approaches, we performed a clustering analysis on the confidence scores provided by STRING. Only clusters with more than five proteins are illustrated, and the nine clusters have the same color notation as their corresponding pathways in Figure S13(a).

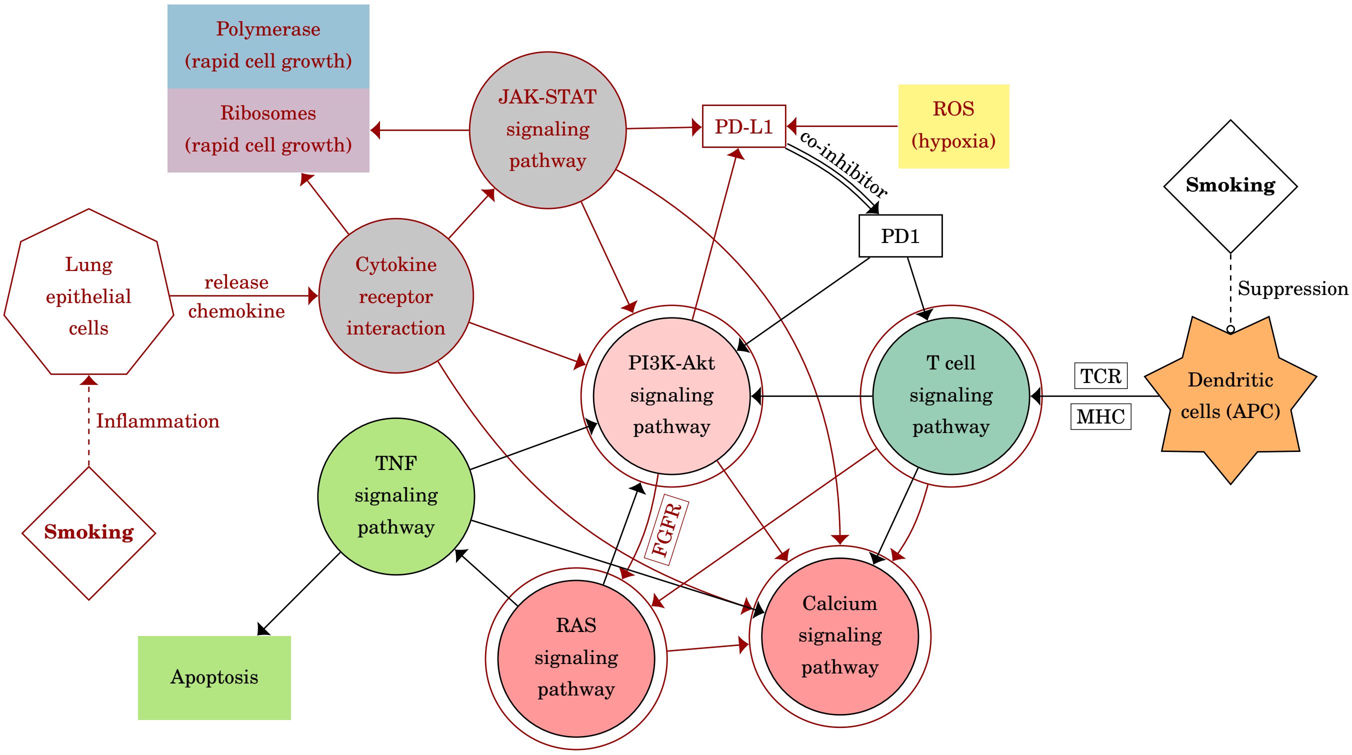

(a) Pathways identified using 1212 genes using the union of decorrelation and multivariate approaches. The color corresponds to the clusters in Figure S12.

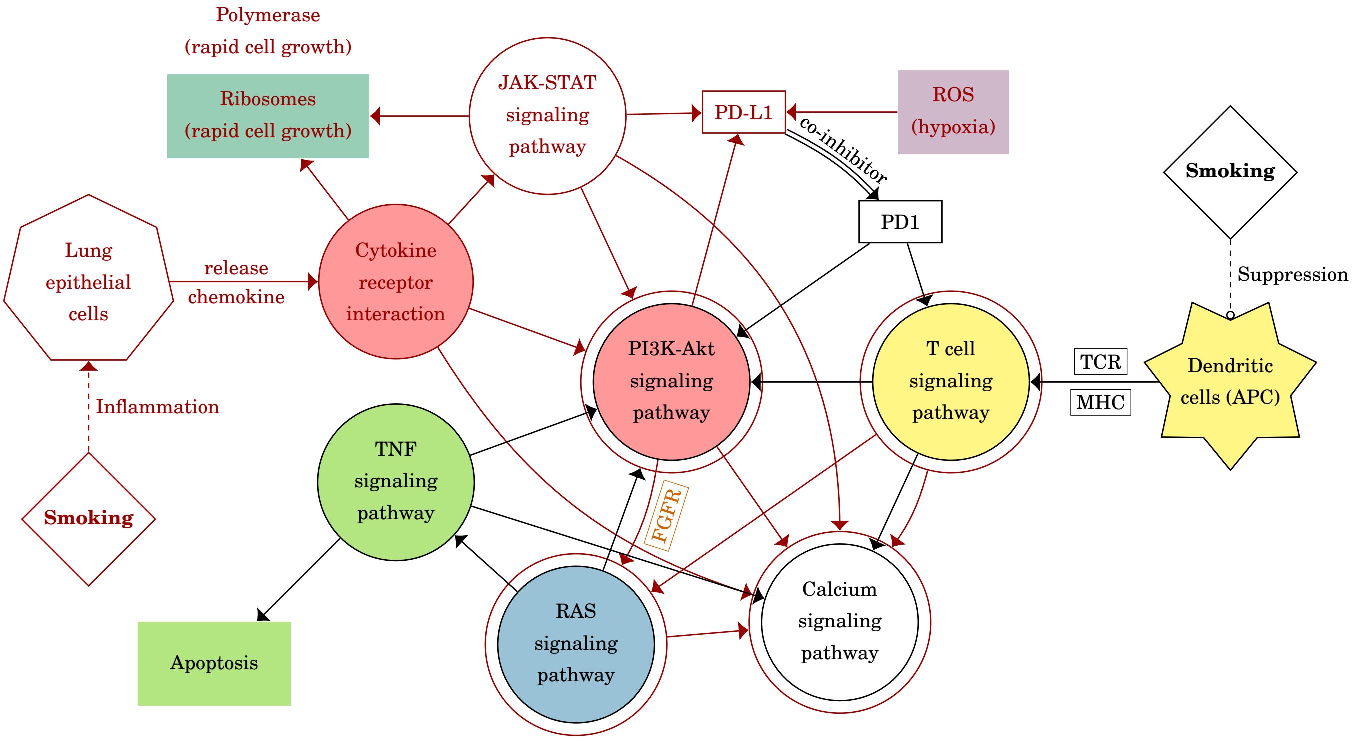

(b) Pathways identified using 590 genes using the hybrid approach. The color corresponds to the clusters in Figure 6.

Figure S13: **Pathway diagram created on the basis of the clustering results.** The circular nodes indicate pathways, the rectangular nodes indicate membrane proteins/receptors, the dark red network indicates the tumor growth direction, and the black network indicates apoptosis.

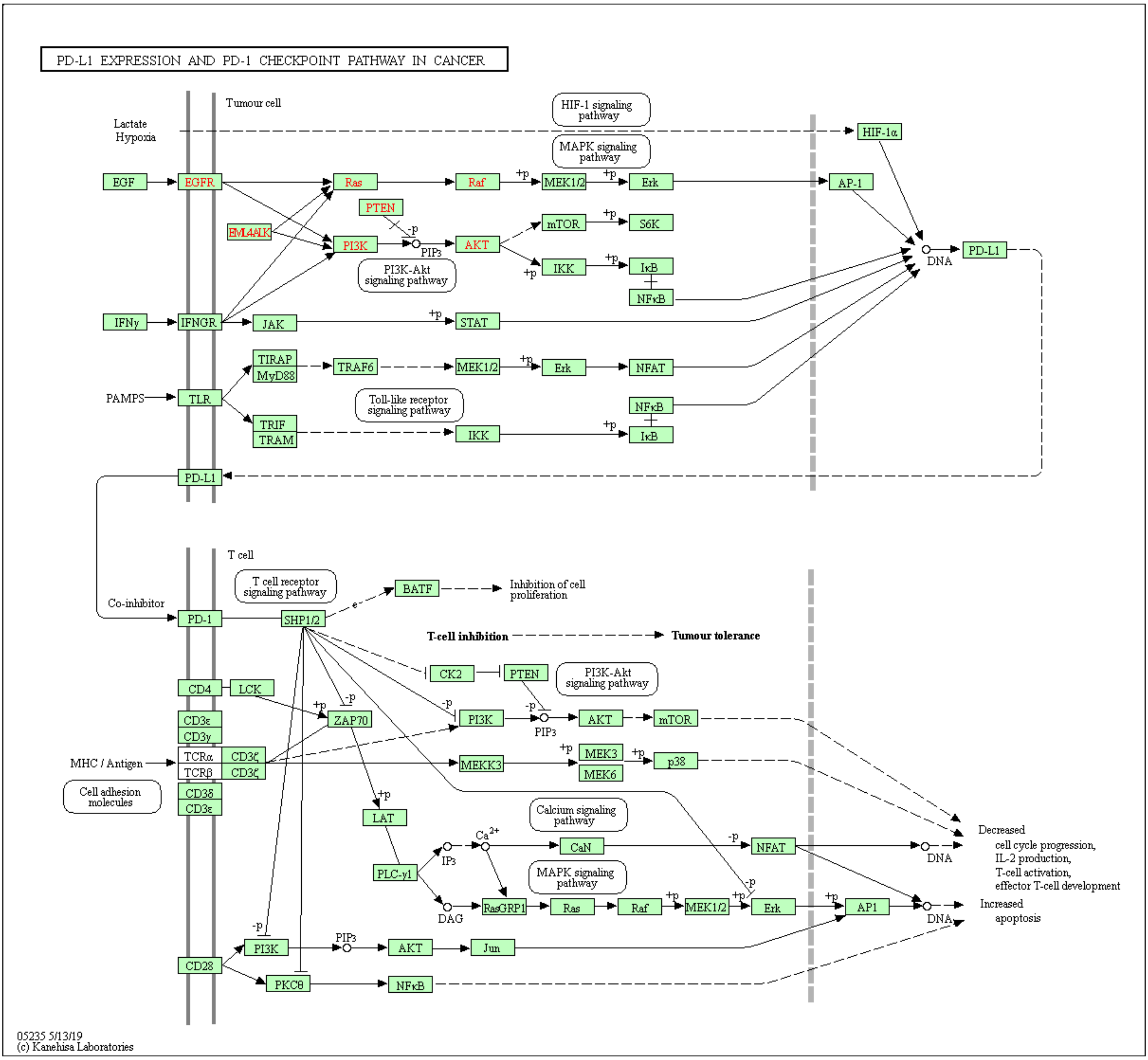

Figure S14: **PD-L1 expression and PD-1 check point in cancer provided by KEGG.** The pathway illustrates the struggle between the apoptosis mechanism and the tumorigenesis mechanism among the T cells and tumor cells. We can observe several key proteins also presented in Figure 6 in the manuscript.

Term ID	Term description	Strength	FDR
hsa05330	Allograft rejection	1.30	5.90e-05
hsa05310	Asthma	1.25	5.70e-04
hsa05332	Graft-versus-host disease	1.21	2.30e-04
hsa04940	Type I diabetes mellitus	1.17	3.00e-04
hsa05320	Autoimmune thyroid disease	1.15	1.20e-04
hsa04672	Intestinal immune network for IgA production	1.13	3.80e-04
hsa00480	Glutathione metabolism	1.04	7.80e-04
hsa05416	Viral myocarditis	1.02	8.80e-04
hsa04658	Th1 and Th2 cell differentiation	1.00	6.85e-05
hsa05321	Inflammatory bowel disease	0.99	1.30e-03
hsa04659	Th17 cell differentiation	0.98	5.90e-05
hsa04612	Antigen processing and presentation	0.96	1.50e-03
hsa04640	Hematopoietic cell lineage	0.93	3.40e-04
hsa05140	Leishmaniasis	0.92	2.10e-03
hsa05145	Toxoplasmosis	0.87	5.90e-04
hsa04380	Osteoclast differentiation	0.80	1.30e-03
hsa05169	Epstein-Barr virus infection	0.78	1.20e-04
hsa03010	Ribosome	0.78	1.60e-03
hsa04514	Cell adhesion molecules	0.75	2.10e-03
hsa05150	Staphylococcus aureus infection	0.75	3.39e-02
hsa05235	PD-L1 expression and PD-1 checkpoint pathway in cancer	0.74	3.55e-02
hsa05164	Influenza A	0.72	1.50e-03
hsa05322	Systemic lupus erythematosus	0.72	4.16e-02
hsa05166	Human T-cell leukemia virus 1 infection	0.70	5.70e-04
hsa05170	Human immunodeficiency virus 1 infection	0.63	5.40e-03
hsa05167	Kaposi sarcoma-associated herpesvirus infection	0.62	1.33e-02
hsa04621	NOD-like receptor signaling pathway	0.59	3.55e-02
SA-5578999	Defective GCLC causes Hemolytic anemia due to gamma-glutamylcysteine synthetase deficiency (HAGGSD)	1.99	3.52e-02
HSA-202430	Translocation of ZAP-70 to Immunological synapse	1.29	4.27e-02
HSA-3299685	Detoxification of Reactive Oxygen Species	1.20	5.90e-04
HSA-936440	Negative regulators of DDX58/IFIH1 signaling	1.07	3.16e-02
HSA-156902	Peptide chain elongation	1.00	2.20e-04
HSA-192823	Viral mRNA Translation	1.00	2.20e-04
HSA-877300	Interferon gamma signaling	1.00	2.20e-04
HSA-2408557	Selenocysteine synthesis	0.98	2.20e-04
HSA-72764	Eukaryotic Translation Termination	0.98	2.20e-04
HSA-2408522	Selenoamino acid metabolism	0.97	4.70e-05

continued on next page

Table S3 – continued from previous page

Term ID	Term description	Strength	FDR
HSA-975956	Nonsense Mediated Decay (NMD) independent of the Exon Junction Complex (EJC)	0.97	2.20e-04
HSA-1799339	SRP-dependent cotranslational protein targeting to membrane	0.94	1.90e-04
HSA-72689	Formation of a pool of free 40S subunits	0.94	2.80e-04
HSA-9633012	Response of EIF2AK4 (GCN2) to amino acid deficiency	0.94	2.80e-04
HSA-156827	L13a-mediated translational silencing of Ceruloplasmin expression	0.91	4.70e-04
HSA-72706	GTP hydrolysis and joining of the 60S ribosomal subunit	0.90	4.70e-04
HSA-975957	Nonsense Mediated Decay (NMD) enhanced by the Exon Junction Complex (EJC)	0.89	5.90e-04
HSA-198933	Immunoregulatory interactions between a Lymphoid and a non-Lymphoid cell	0.83	1.20e-03
HSA-913531	Interferon Signaling	0.81	1.30e-04
HSA-9010553	Regulation of expression of SLITs and ROBOs	0.76	1.30e-03
HSA-6791226	Major pathway of rRNA processing in the nucleolus and cytosol	0.73	2.20e-03
HSA-1280215	Cytokine Signaling in Immune system	0.58	4.29e-06
HSA-449147	Signaling by Interleukins	0.52	5.10e-03
HSA-71291	Metabolism of amino acids and derivatives	0.50	3.16e-02
HSA-2262752	Cellular responses to stress	0.48	4.90e-03
HSA-1280218	Adaptive Immune System	0.46	1.20e-03
HSA-168256	Immune System	0.42	1.26e-07
HSA-168249	Innate Immune System	0.34	2.87e-02
HSA-1643685	Disease	0.29	2.10e-02
GO:0002587	Negative regulation of antigen processing and presentation of peptide antigen via mhc class ii	1.99	4.88e-02
GO:0097069	Cellular response to thyroxine stimulus	1.99	4.88e-02
GO:1904172	Positive regulation of bleb assembly	1.99	4.88e-02
GO:2001188	Regulation of t cell activation via t cell receptor contact with antigen bound to mhc molecule on antigen presenting cell	1.69	1.13e-02
GO:0002578	Negative regulation of antigen processing and presentation	1.51	2.24e-02
GO:0071372	Cellular response to follicle-stimulating hormone stimulus	1.42	3.26e-02
GO:0002507	Tolerance induction	1.38	3.72e-02
GO:0001916	Positive regulation of t cell mediated cytotoxicity	1.19	2.18e-02
GO:0043368	Positive t cell selection	1.16	2.65e-02
GO:2000108	Positive regulation of leukocyte apoptotic process	1.11	3.52e-02
GO:0002230	Positive regulation of defense response to virus by host	1.08	4.05e-02
GO:0071354	Cellular response to interleukin-6	1.07	4.34e-02

continued on next page

Table S3 – continued from previous page

Term ID	Term description	Strength	FDR
GO:0060333	Interferon-gamma-mediated signaling pathway	1.04	3.50e-04
GO:0032480	Negative regulation of type i interferon production	1.03	1.76e-02
GO:0006613	Cotranslational protein targeting to membrane	0.99	6.85e-05
GO:0045454	Cell redox homeostasis	0.99	8.40e-03
GO:0006614	SRP-dependent cotranslational protein targeting to membrane	0.96	3.50e-04
GO:2000106	Regulation of leukocyte apoptotic process	0.95	1.20e-03
GO:0050671	Positive regulation of lymphocyte proliferation	0.94	1.59e-05
GO:0070228	Regulation of lymphocyte apoptotic process	0.94	3.56e-02
GO:0072599	Establishment of protein localization to endoplasmic reticulum	0.93	1.70e-04
GO:0014823	Response to activity	0.93	1.49e-02
GO:0042102	Positive regulation of t cell proliferation	0.90	2.30e-03
GO:0001910	Regulation of leukocyte mediated cytotoxicity	0.90	1.86e-02
GO:0019083	Viral transcription	0.88	1.10e-03
GO:0045582	Positive regulation of t cell differentiation	0.88	8.40e-03
GO:0000184	Nuclear-transcribed mrna catabolic process, nonsense-mediated decay	0.86	1.40e-03
GO:0032652	Regulation of interleukin-1 production	0.84	1.40e-02
GO:0032088	Negative regulation of nf-kappab transcription factor activity	0.83	3.19e-02
GO:0050870	Positive regulation of t cell activation	0.81	3.13e-05

Table S3: **Cluster annotation table provided by the STRING.** We listed significant *KEGG pathway*, *Reactome Pathway*, and the top 30 *GO Biological Process* provided by STRING for the annotation of the methyl-gene dataset. This annotation is based on the union gene set provided by the decorrelation and multivariate approaches.

References

[1] Yen-Tsung Huang. Genome-wide analyses of sparse mediation effects under composite null hypotheses. *The Annals of Applied Statistics*, 13(1):60–84, 2019.

[2] Richard Barfield, Jincheng Shen, Allan C Just, Pantel S Vokonas, Joel Schwartz, Andrea A Baccarelli, Tyler J VanderWeele, and Xihong Lin. Testing for the indirect effect under the null for genome-wide mediation analyses. *Genetic epidemiology*, 41(8):824–833, 2017.

[3] Yen-Tsung Huang and Wen-Chi Pan. Hypothesis test of mediation effect in causal mediation model with high-dimensional continuous mediators. *Biometrics*, 72(2):402–413, 2016.

[4] Naftali Weinberger. Faithfulness, coordination and causal coincidences. *Erkenntnis*, 83(2):113–133, 2018.

[5] Yen-Tsung Huang. Variance component tests of multivariate mediation effects under composite null hypotheses. *Biometrics*, 75(4):1191–1204, 2019.

[6] Mohamed Mounir, Marta Lucchetta, Tiago C Silva, Catharina Olsen, Gianluca Bontempi, Xi Chen, Houtan Noushmehr, Antonio Colaprico, and Elena Papaleo. New functionalities in the tcgabiolinks package for the study and integration of cancer data from gdc and gtex. *PLoS computational biology*, 15(3):e1006701, 2019.

[7] Robert L Grossman, Allison P Heath, Vincent Ferretti, Harold E Varmus, Douglas R Lowy, Warren A Kibbe, and Louis M Staudt. Toward a shared vision for cancer genomic data. *New England Journal of Medicine*, 375(12):1109–1112, 2016.

[8] Michael A Gillette, Shankha Satpathy, Song Cao, Saravana M Dhanasekaran, Suhas V Vasaikar, Karsten Krug, Francesca Petralia, Yize Li, Wen-Wei Liang, Boris Reva, et al. Proteogenomic characterization reveals therapeutic vulnerabilities in lung adenocarcinoma. *Cell*, 182(1):200–225, 2020.
